# Detecting past changes of effective population size

**DOI:** 10.1111/eva.12170

**Published:** 2014-06-16

**Authors:** Natacha Nikolic, Claude Chevalet

**Affiliations:** 1 IFREMER La RéunionLe Port, France; 2 INRA Génétique, Physiologie et Systèmes d'ElevageCastanet-Tolosan, France; 3 Université de Toulouse INP, ENSAT, Génétique, Physiologie et Systémes d'ElevageCastanet-Tolosan, France; 4 Université de Toulouse INP, ENVT, Génétique, Physiologie et Systémes d'ElevageToulouse, France

**Keywords:** Atlantic salmon, effective population size, microsatellites, past demography.

## Abstract

Understanding and predicting population abundance is a major challenge confronting scientists. Several genetic models have been developed using microsatellite markers to estimate the present and ancestral effective population sizes. However, to get an overview on the evolution of population requires that past fluctuation of population size be traceable. To address the question, we developed a new model estimating the past changes of effective population size from microsatellite by resolving coalescence theory and using approximate likelihoods in a Monte Carlo Markov Chain approach. The efficiency of the model and its sensitivity to gene flow and to assumptions on the mutational process were checked using simulated data and analysis. The model was found especially useful to provide evidence of transient changes of population size in the past. The times at which some past demographic events cannot be detected because they are too ancient and the risk that gene flow may suggest the false detection of a bottleneck are discussed considering the distribution of coalescence times. The method was applied on real data sets from several Atlantic salmon populations. The method called *VarEff* (Variation of Effective size) was implemented in the R package *VarEff* and is made available at https://qgsp.jouy.inra.fr and at http://cran.r-project.org/web/packages/VarEff.

## Introduction

The results from genetic surveys may be used to infer the demographic history of species and populations, and may help to make conservation management decisions. Analyzing the distribution of DNA polymorphism at several genetic markers has become the basis for inferring relationships between individuals or groups of individuals, and has been extensively used to derive estimations of the time since divergence between species or populations. Coalescence theory and the development of Bayesian approaches have made it possible to take advantage of the complete information available in samples of alleles drawn in populations and to derive estimates of various parameters. One of the main achievements was the possibility to obtain information on the past history of populations, especially in the case of human populations (Shriver et al. 1997; Reich and Goldstein 1998). The coalescent process introduced by Kingman (1982a,b) provides a mathematical framework which describes the distribution of gene trees in populations and helps derive evolutionary relationships. The inheritance relationships between alleles are represented as a gene genealogy known as the coalescent. Coalescence theory considers a sample of genes from a population to trace all alleles to a single ancestral copy, named as the Most Recent Common Ancestor (MRCA). Several approaches based on coalescence theory and tools from computational statistics have been developed in the late 1980s: the moment-matching approaches (Slatkin and Hudson 1991; Rogers and Harpending 1992; Rogers 1995; Shriver et al. 1997), population decline and growth detection (Cornuet and Luikart 1996; Weiss and von Haeseler 1998), and likelihood approaches with varying population size (Griffiths and Tavaré 1994; Kuhner et al. 1998). When sequence data are available (Drummond et al. 2005) building the coalescence tree of the sampled alleles allows branch lengths of the tree to be estimated, hence the effective population size from the mutation rate. It also provides a ‘Bayesian skyline plot’ estimating past population dynamics through time from a sample of molecular sequences (Drummond et al. 2005) or from complete genome sequences of a few individuals (Li and Durbin 2011). However, genome sequencing remains very expensive, and less available than microsatellites analysis for most nonmodel species. Inferring demographic events from microsatellites data was considered by Wilson and Balding (1998), Beaumont (1999), and more recently by Wu and Drummond (2011). Applying the ‘skyline plot’ approach to microsatellite polymorphism at dozens of loci presents several difficulties because the mutational process of microsatellites only provides poor information on the coalescence trees, and the calculation of the exact likelihood needs the simulation of very many admissible trees which requires very long calculation time (e.g., *MSVAR* software). The Approximate Bayesian Computation (ABC) approach was also proposed to investigate population history from current genetic data (Cornuet et al. 2008; Hoffman et al. 2011), but the incidence of priors seemed stronger using ABC than classical Bayesian method in the case of unreliable field data that may suggest to set priors far from reality (Nikolic et al. 2009b).

Here, we address the question using microsatellites and an approximation of likelihood based on the distribution of distance frequencies *f*_*k*_ between alleles where *f*_*k*_ is the frequency of pairs of alleles differing by *k* microsatellite motifs. This distribution could be characterized in the case of a variable past effective population size (Chevalet and Nikolic 2010). This allowed a new approach to be proposed, which provides different views of the posterior distribution of past effective population size (means, mode, median, and quantiles) as well as the complete posterior distribution at some times. Also, it allows the posterior distribution of the Time to the MRCA between two alleles (*T*_MRCA_) to be recovered. This property was used to discuss the risk that a false bottleneck be detected in a population submitted to immigration, comparing the expected distributions of *T*_MRCA_ under both hypotheses. The method was evaluated and discussed in comparison with *MSVAR* (Beaumont 1999) which makes use of the same type of data. It was implemented into an R package *VarEff*, available at http://cran.r-project.org/web/packages/VarEff and at https://qgsp.jouy.inra.fr.

## Materials and methods

We detail the genetic and demographic framework used in the present study, and outline the statistical setting used, assuming the studied population remained isolated. In order to discuss the effects of gene flow on the results, we developed a simple model to illustrate how permanent immigration may mimic the effect of a recent bottleneck on the distribution of *T*_MRCA_. Details on implementation and its uses can be found in the *VarEff* package and at the Quantitative Genetics Software Platform (https://qgsp.jouy.inra.fr).

### Genetic diversity at microsatellites markers

#### Mutation models

We consider a general symmetrical Stepwise Mutation Model, allowing the number of microsatellite motifs to be changed under mutation by +*r* or −*r* with probability *m*_*r*_. This process is defined by the mutation rate *μ* and by the characteristic function *M*(*x*) = Σ_*r* > 0_ *m*_*r*_ cos (*rx*). For the Single Step Mutation model (SSM), *m*_1_ = 1, and:


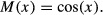
(1)

Two other models are considered, needing an additional parameter *c* < 1 to fix them: a special case of the Two Phase model (Di Rienzo et al. 1994) in which a proportion *c* of the mutational events gives rise to a variation of two motifs so that *m*_1_ = 1−*c* and *m*_2_ = *c*, and the Geometrical Stepwise Mutation model (Whittaker et al. 2003; Watkins 2006) for which *m*_*r*_ = (1 − *c*)*c*^*r*−1^.

#### Transformation of data

At any microsatellite locus, the observed diversity is given by a list of alleles in a sample. Each allele is characterized by the length of an amplified DNA fragment and is named *i*, the number of repeats in excess relative to the shortest allele of the sample. A sample of *n* alleles is described by a list *n*_0_, *n*_1_, *n*_2_,…, *n*_*i*_,… where *n*_*i*_ is the number of alleles with *i* repeat motifs. Pure coalescence-based methods use this complete information (*MSVAR*, Beaumont 1999). Instead, we used a transformed version of the data made up of the frequencies of pairs of alleles at a given distance (Shriver et al. 1997), i.e., the quantities 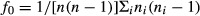 and 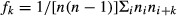 for *k* ≠ 0. Theoretically, there is no one-to-one correspondence between the lists (*n*_*i*_) and (*f*_*k*_), but in practice (using actual diversity data) a single list of *n*_*i*_'s values could be found to fit the *f*_*k*_'s. Global estimates of effective size can be derived from homozygosity *f*_0_,


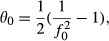
(2)

and from the first two moments of *f*_*k*_'s (Pritchard and Feldman 1996; Chevalet and Nikolic 2010):


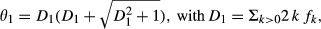
(3)


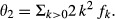
(4)

### Modeling population size changes

In order to cope with any kind of population size variation, not only to continuous growth or decrease, we chose to model past changes of population size by step functions (‘skyline plots’), so that the size remains equal to *N*_*i*_ in successive time intervals [*g*_*i*_, *g*_*i* + 1_], 0 ≤ *i* ≤ *J*−1 (Pybus et al. 2000). In this setting, *g*_0_ = 0 and *N*_0_ stand for the present time and the current population size, *g*_*i*_ < *g*_*i* + 1_ and *N*_*J*_ stands for an ancestral population size, assumed constant for times older than *g*_*J*_. In the course of calculations, time scale is changed from generation number to *τ* = *gμ* where *g* is generation number and *μ* the mutation rate, and population sizes are normalized as *θ* = 4 *Nμ* values. A demographic history is characterized by 2*J* + 1 parameters, i.e., the *J* + 1 values,


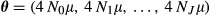


and the *J* time intervals


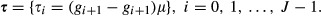


In the process of estimating past history, such step functions are randomly generated and the likelihood of data is calculated conditional on the mutation process and on the demographic hypothesis.

### Approximate likelihood

For a given ‘skyline plot’ demographic history defined by parameters (***θ***, ***τ***), Chevalet and Nikolic (2010) showed how the probability that two microsatellite alleles differ by *k* motifs can be rapidly calculated through a numerical integration (a summary of the rationale of this result is given in Data S1). Assuming that the *L* chosen markers are genetically independent and are submitted to the same mutational process, the vector 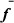 of mean values of frequencies *f*_*k*_ at the different loci is expected to approximately follow a multinormal distribution with means 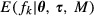 and covariance matrix 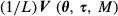, where the moments are conditioned on the past demography (***θ***, ***τ***) and on the mutation process (function *M*). The likelihood of data was then approximated from this distribution of the mean values of the observed *f*_*k*_ at the different loci. An un-normalized expression of approximate likelihood is then reduced to a quadratic:


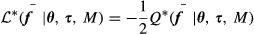


with:


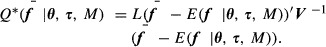
(5)

Choosing a fixed range [0, *d*_*f*_] of *k* distances, the expectation 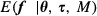 of the vector 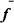 of mean values depends in a calculable way on the parameters ***θ*** and ***τ*** and on the mutation model. Calculating the matrix ***V***(***θ***, ***τ***, *M*) of variances and covariances of the *f*_*k*_'s under the same conditions would require much computation time. Hence, the model was over-simplified, assuming that ***V***(***θ***, ***τ***, *M*) depended weakly on parameters and could be replaced by a constant matrix, based on its sample estimate. In eqn (5), ***V*** was taken as a constant


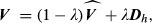
(6)

where 0 < *λ* < 1, 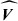 is the sample estimate and ***D***_*h*_ a diagonal matrix made up of a heuristic approximation of *f*_*k*_ variances (Chevalet and Nikolic 2010),


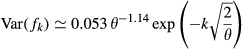


based on the *θ*_1_ estimate of *θ* eqn (5).

Approximate normality is expected from the law of large numbers, but convergence may be slow. Using simulated demographic scenarios, approximate normality was indirectly assessed, testing the distribution of the quadratic form eqn (5) against the Chi-square distribution with (*d*_*f*_ + 1) degrees of freedom. In each set of 100 simulated cases, ***V*** was estimated from the whole data set and used as the true ***V*** matrix. Then frequencies in each simulation (*i*) were used to calculate the corresponding ***Q***_*i*_ value, and the distribution of the 100 ***Q***_*i*_ values was tested against the corresponding Chi-square distribution, using the Kolmogorov–Smirnov test. The corresponding *P*-values are given in Table 1.

**Table 1:** Test of the normality of mean allele distance frequencies. *P*-values of the Kolmogorov–Smirnov test of the distribution of the quadratic forms *Q* eqn (5) against the Chi-square distribution with *d*_*f*_ + 1 degrees of freedom, which is expected if the mean frequencies of allele distances follows a multinormal distribution. In the 14 simulated cases described in Figs 2–4, *P*-values were calculated from data obtained with 10, 20 and 40 markers, using the *ks*.*test* function available in the R package

Case	References	*d*_*f*_ + 1	*L* = 40	*L* = 20	*L* = 10
Constant population size	Fig. 3A	20	0.142	0.227	<0.001
Population expansion	Fig. 2A	10	0.740	0.169	0.239
	Fig. 2B	12	0.623	0.114	0.022
	Fig. 2C	10	0.192	0.134	<0.001
	Fig. 2D	12	0.402	0.124	<0.001
	Fig. 2E	10	0.056	0.007	<0.001
	Fig. 2F	12	0.058	0.004	<0.001
Present or past bottleneck	Fig. 3B	15	0.110	0.207	0.013
	Fig. 3C	15	0.158	0.035	0.001
	Fig. 3D	15	0.712	0.116	0.011
	Fig. 3E	15	0.850	0.214	0.822
	Fig. 3F	15	0.644	0.574	0.021
Transient increase in the past	Fig. 4A	15	0.492	0.214	0.323
	Fig. 4B	15	0.528	0.051	0.153

### Statistical inference and implementation of the method

Using the approximate expression of likelihood, inference was based on a Metropolis Hastings Bayesian scheme. Prior means of *θ*'s are set equal to a single value *θ*_*p*_ given by the user, from a single prior value *N*_*p*_ of effective size and an assumed mutation rate *μ*. Since the model is expressed with functions of the compound parameters ***θ*** and ***τ***, the mutation rate is fixed and behaves as a scale parameter. The prior distribution of ***θ*** is assumed to be a multinormal distribution on the logarithmic scale, characterized by a single variance. Following a suggestion of Drummond et al. (2005), correlations *ρ*^*k*^ between ln(*θ*_*i*_) and ln(*θ*_*i* + *k*_) can be introduced in order to avoid too large variations between successive population sizes. Prior means of ***τ*** are set equal to a single value, equal to *τ*_*p*_ = *g*_*J*_*μ*/*J* where *g*_*J*_ is the number of generations since the population departed from an assumed ancestral size. The prior mean of g_*J*_ must be given by the user. As for *θ*'s, a normal prior distribution is assumed for the logarithms of the *τ*'s, with another single variance and independence between time intervals. For the joint prior distribution, independence is assumed between ***θ*** and ***τ***. Denoting with ***π*** the set of log-parameters ln(***θ***/*θ*_*p*_) and ln(***τ***/*τ*_*p*_), and with ***W*** their (2*J* + 1) × (2*J* + 1) prior variance-covariance matrix, the prior probability of a set of parameters is then written as


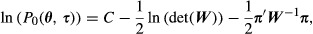


noting that the special form of ***W*** makes these calculations simple and fast. Combining with eqn (5), an un-normalized expression of the log-posterior probability of parameters is:


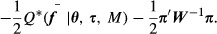


Statistical inference was performed using the Metropolis algorithm based on this expression and using normal proposal distributions for the logarithms of parameters as follows. The move of parameters values from the *u*th to the (*u* + 1)th iteration is obtained as:


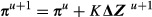


where ***π***^*u*^ stands for the current values, ***Z***^*u* + 1^ is a random vector of normal standard variates with zero mean and covariance matrix equal to the identity matrix, ***Δ*** is the matrix such that ***W*** = ***ΔΔ***′ is the Choleski decomposition of ***W***, and *K* is a scale factor used to adjust the acceptance rate at a desired value. Implementation of the Metropolis-Hastings algorithm made use of the *metrop* function of the *mcmc* library (Geyer 2009) available in the R environment (R Development Group Team 2008, version R 2.10.10 or later).

The method was implemented in an R package available at http://cran.r-project.org/web/packages/VarEff. At the end of a run of *VarEff*, a list of demographic step functions is generated. Each one is described by *J* steps characterized by population sizes *θ*_*j*_ (0 ≤ *j* ≤ *J*) and times of size changes *τ*_*j*_. This allows the posterior distribution of past effective size to be recovered at any time in the past. The functions provided in the package allow the user to visualize these distributions at different times in the past, to extract global statistics from these distributions (arithmetic and harmonic means, mode, median, and quantiles) and to derive the posterior distribution of the *T*_MRCA_ of two random alleles (eqn A2, Data S1).

### Detecting changes of past population size

A global criterion, the imbalance index (*i* = ln *θ*_2_ – ln *θ*_0_, Kimmel et al. 1998), was used to check population size changes. In addition, we devised a new criterion based on the estimated population sizes in the past, during some interval of time. Using estimates 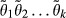 of population size at several times in some period, we considered the ratio (RN) of the range of estimated population sizes during the period, to the arithmetic mean of 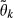
's:


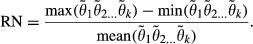
(7)

In the following, RN values were based on the medians of posterior distributions.

### Modeling the effects of gene flow

Confounding effects between recent population size decrease (bottleneck) and gene flow has been reported several times. To help understand how both phenomena may affect estimation of population size changes, we considered the incidence of a simple migration model on the distribution of the *T*_MRCA_ of two alleles. Calculations are detailed in Data S2. For an isolated population, there is a direct link between the function describing the change with time of population size and the distribution of the *T*_MRCA_ of two alleles (eqn A2 in Data S1). Plugging in it the expected distribution of *T*_MRCA_ under immigration (eqn A3 in Data S2) generates a function *θ*(*τ*) describing a change with time of population size. Assuming the sampled population has a constant effective size *N*, but receives immigrants at a rate *m* each generation from a much larger population of size *N*/*ε*, one would predict from eqn (A5) (Data S2) that it underwent a bottleneck *g*_*b*_ generations ago, with:


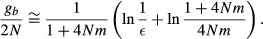
(8)

### Data sets

#### Simulated data sets

Several scenarios of population demography were simulated to test the efficiency of the method at estimating effective population size and at detecting past demographic variations. As a rule, samples of 40 diploid individuals were drawn at different times when the analysis was performed. In general, 40 independent microsatellite markers were generated according to the Single Step Model; simulated population sizes were in the range between 100 and 10 000 and mutation rates were adjusted between 0.01 and 0.001 to reach the considered *θ* values. Main scenarios were replicated 100 times, so as to account for the effects of drift on the precision of estimators and to allow comparison with standard ones for constant population size. Details about scenarios are given in the legends of Figures. An in-house forward software was used, that allows population size changes and gene flow between several populations to be simulated, and that makes it possible to consider various stepwise mutation models.

#### Atlantic salmon data sets

We used the genetic data set analyzed by Nikolic et al. (2009a), composed of 367 wild adult anadromous Atlantic salmon (i.e., adults migrate from the sea to breed in freshwater) from North-West France (Oir and Scorff) and North-East of Scotland (Spey and Shin) sampled in 2005 and earlier in 1988 except for Shin sampled in 1992. These individuals have been genotyped with 37 nuclear microsatellites and the mutation rate detected was 0.0003 (Nikolic et al. 2009b). Concerning the census sizes we used the ones reported in Nikolic et al. (2009a).

## Results

We first provide technical results on the behavior of the algorithm implemented in *VarEff* (choice of priors, convergence). Then, we evaluate the efficiency of the method to estimate past demography in cases when population size has undergone transient changes and compare it with *MSVAR*. Most results were derived from a set of simulated data, as described in the Figures. Finally, we apply the method to the salmon data set.

### Technical considerations

#### Tuning parameters and priors

Running the MCMC chain requires tuning some parameters and checking the effects of priors. Prior values are required to set the range of admissible parameter values. Since the global *θ* estimates give the order of magnitude of population size, the prior for population size (*N*_*p*_) must be adjusted to the given mutation rate. We propose to set *N*_*p*_ equal to *θ*_1_/(4*μ*), since *θ*_1_ eqn (3) generally takes an intermediate value between *θ*_0_ and *θ*_2_ eqns (2) and 4). Choosing a valuable time horizon (*g*_*J*_) is also important. It is the time before which population size is assumed constant in the model. Choosing it too small would prevent the method to search for ancient variations and cause biases for recent sizes. Prior knowledge about the history of the population must be used to set a reasonable *g*_*J*_ value. For the population size and the time intervals between jumps of the step functions, a variance must be given to fix the prior distribution of the logarithms of these parameters. A value of 3 turned out to be a good choice for both parameters. This value allows for searches with 20- to 40-fold relative variations of population sizes and time intervals. Larger values may prevent the algorithm to converge.

Some other parameters must be fixed: they are not subject to estimation but may have some impact on the efficiency of the method. We found that computing time was roughly proportional to the product J*d_*f*_ where *J* is the number of population size changes and *d*_*f*_ is the largest difference between allele lengths. Trials run with small *J* (between 2 and 5) or large *J* (>10) did not prove that using larger values provided better results. Since the calculation time is proportional to *J*, we found that it was more efficient to run simulations with a limited number of population size changes (*J* = 4–5) than to use large *J* values. The range *d*_*f*_ of allele distances must include significant distances found in the sample showing for example a frequency larger than 0.005. Including the largest distances may be useful to detect past events, but often leads to include distances with zero frequency, a case that makes unrealistic the assumption of normality of the mean frequency distribution. A correlation *ρ* between the successive population sizes generated by the proposal distribution must be given. Trials did not prove this parameter to be of main importance, except if a large value of the number of steps *J* is used. Using a large *ρ* prevents the method to search for large variations of past effective size. Using a very large value (*ρ* = 0.99) is a way to constraint the method to search for a constant population size. A heuristic parameter *λ* is introduced in the calculation of an approximate likelihood, to balance the observed covariance structure by a theoretical diagonal variance matrix eqn (16) and avoid numerical instability. The effect of this parameter on the accuracy of estimations was checked in the case of constant population size. Such a result is illustrated in the Figure S1, for the mode and median estimates of population size, and it suggests using for *λ* a value near 0.5.

#### Complexity and convergence

The complexity of the algorithm is roughly proportional to the product J*d_*f*_, and is independent of the number *L* of markers and of sample size. In general, convergence seemed to be obtained with *metrop* (Geyer 2009) parameters (*nbatch* = 10 000, *blen* = 1, *nspac* = 10) after a burnin period of 10 000 steps. Smoother results were obtained, averaging results over several steps (*blen* = 10). However, it may be better to increase *nspac*, the space between sampled states, to 100 or 500, so as to get rid of autocorrelations between steps. Convergence was assessed comparing several series of simulations with the test of Gelman and Rubin (1992). This test can be applied to the series of (***θ***, ***τ***) values output from the *VarEff* function, or to the series of estimated population sizes at a number of times.

The time needed to get a sound result, using *metrop* indices (10 000, 10, 10) or (10 000, 1, 100) for a total of 10^6^ steps was about 90–160 min on a PC (Intel Core2 Quad CPU Q6600 at 2.40 Ghz processor, Windows operating system). This makes our method much faster than *MSVAR*, which is dependent on the number of markers. For example (Table 2), it took about 10–16 h with the same computer to analyze the same data sets with rather short chains (20 000 output lines and 10 000 iterations between them).

**Table 2:** Comparison of *MSVAR* and *VarEff* results. *MSVAR* and *VarEff* were run on the same data, for six cases corresponding to population expansion and to recent or ancient transient past decrease or increase of population size, as defined in the mentioned Figures. *N*_0_ stands for size at sampling time, *N*_*a*_ for the ancestral size and *T*_f_ for the time since the beginning of population size changes (denoted also as *g*_*J*_ in the Materials and methods section). For nonmonotone history, *N*_*i*_ and time stand for an intermediate population size and the corresponding time (generation number)

Case	*N*_0_	*N*_*i*_ (time)	*N*_*a*_	*T*_*f*_	Calculation time
Current expansion					
Fig. 2C					
Theory	5000	NA	500	300	
*MSVAR*	5400	NA	410	415	12 h 35 min
*VarEff*	2100	NA	540	680	1 h 10 min
Past expansion					
Figs 2D and 5					
Theory	5000	5000 (700)	500	2500	
*MSVAR*	6100	NA	290	2500	12 h 20 min
*VarEff*	5500	5000	630	2000	1 h 25 min
Recent bottleneck					
Figs 3C and 6C					
Theory	1000	100 (250)	10 000	300	
*MSVAR*	1300	NA	625	2	10 h 30 min
*VarEff*	950	260 (260)	1450	1000	2 h 40 min
Past bottleneck					
Figs 3D and 6D					
Theory	1000	100 (450)	1000	500	
*MSVAR*	1070	NA	590	140	15 h 30 min
*VarEff*	1380	270 (400)	1500	1500	2 h 40 min
Recent transient increase					
Fig. 4B					
Theory	500	2500 (350)	500	600	
*MSVAR*	330	NA	870	9	10 h
*VarEff*	570	1480 (420)	400	1500	1 h 30 min
Ancient transient increase					
Fig. 4D					
Theory	500	2500 (650)	500	900	
*MSVAR*	400	NA	710	220	13 h
*VarEff*	520	1600 (810)	640	2500	1 h 35 min

### Efficiency and detection of population size changes

#### Relative efficiency of global size estimates (constant population size)

**Figure 1:**
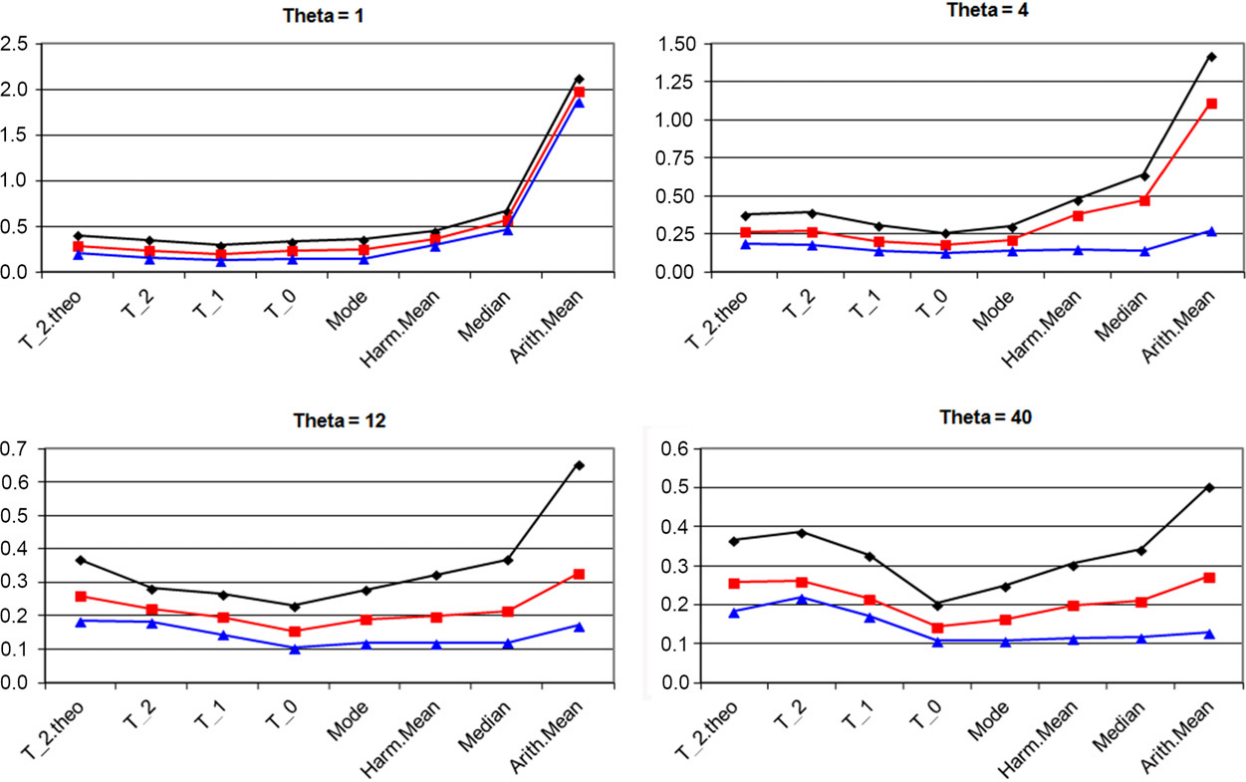
Efficiencies of population size estimates. Efficiencies of seven population size estimates, as function of the genetic diversity (*θ* = 1, 4, 12, and 40) and of the number of markers (10, 20, and 40). Abscissa: T_0, T_1, and T_2 indicate the precision of the global estimates *θ*_0_, *θ*_1_, and *θ*_2_ based on the analysis of 100 replicates of the drift process, T_2.theo provides the theoretical accuracy of *θ*_2_. Mode, Harm.Mean, Median, and Arith.Mean, respectively, stand for the averages of the mode, the harmonic mean, the median and the arithmetic mean of the posterior distributions of past effective population size at 10 times in the past, in the range 0–0.25 (reduced time unit). Ordinates: Efficiencies are measured as the ratios 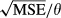 of the square root of the Mean Square Error (MSE) of the estimate to the true *θ* value. Black diamonds: 10 markers; Red squares: 20 markers; Blue triangles: 40 markers.

Simulated data were generated under the simplest case of constant effective population size. The diploid population size was set to 1000, the mutation rate adjusted to get *θ* values of 1, 4, 12, and 40, and simulations were performed according to the Single Step Mutation model for microsatellites (SSM model, eqn 1). For each run, seven estimates of *θ* were derived: three global estimates eqns (2)–(4) and four estimates of population size at 10 times in the past. At each time, four statistics were derived from the posterior distribution of population size: the arithmetic and harmonic means, the mode, and the median. The Mode estimate, for example, was the average of the modes of the posterior distributions of *θ* at times 0.025, 0.05, …, 0.225 and 0.25 in the past (reduced time). Efficiencies were measured as the ratio 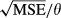 of the square root of the Mean Square Error of the estimate to the true *θ* value (Fig. 1). These efficiencies can be compared to that of *θ*_2_ eqn (4), for which the SSM theory provides the expected value (Pritchard and Feldman 1996):


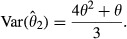
(9)

The Figure S2 illustrates the behavior of estimates at several times in the past, when the population size is constant with *θ* = 40.

#### Detecting past variations of population size

**Figure 2:**
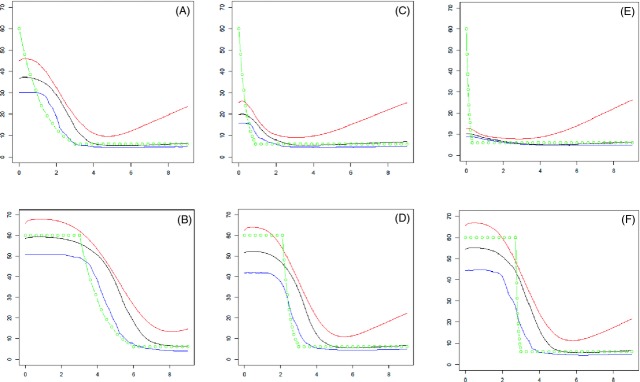
Estimates of population size under expansion. Cases A, C, and E: estimates of current and past population size during expansion. Cases B, D, and F: estimates of current and past population size, from samples performed some time after expansion had finished. Cases A and B: slow expansion, from size 500 to 5000 in 900 generations. Cases C and D: medium expansion rate, from size 500 to 5000 in 270 generations. Cases E and F: fast expansion, from size 500 to 5000 in 90 generations. The 100 simulations were run using a mutation rate of 0.003 and estimations were based on 40 independent markers. Abscissa: time in the past from 0 to 9 (reduced time scale). Ordinates: estimates of past population sizes (theta scale) from arithmetic means (red), medians (black) and modes (blue) of posterior distributions. The simulated demography is shown in green.

**Figure 3:**
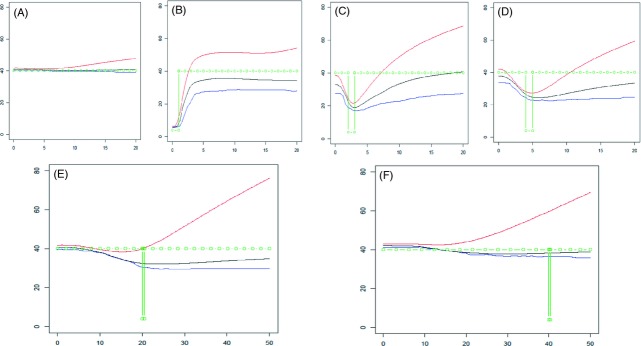
Estimates of population size after a bottleneck. Populations were simulated assuming a population size of 1000 except during a bottleneck with population size reduced to 100 during 100 generations. Mutation rate was set to 0.01, so that *θ* values were equal to 40 except in the bottleneck stage when it was 4. The population was analyzed before the bottleneck (case A), at the end of the bottleneck (case B) and at times 2, 4, 20, and 40 after the bottleneck (cases C–F). Estimations were based on 40 independent markers. Abscissa: time in the past, in reduced scale generation × mutation rate, from 0 to 20 in cases A–D, and from 0 to 50 in cases E and F. Ordinates: estimates of past population sizes (in the reduced *θ* scale) from arithmetic means (red), medians (black), and modes (blue) of posterior distributions. The simulated demography is shown in green.

**Figure 4:**
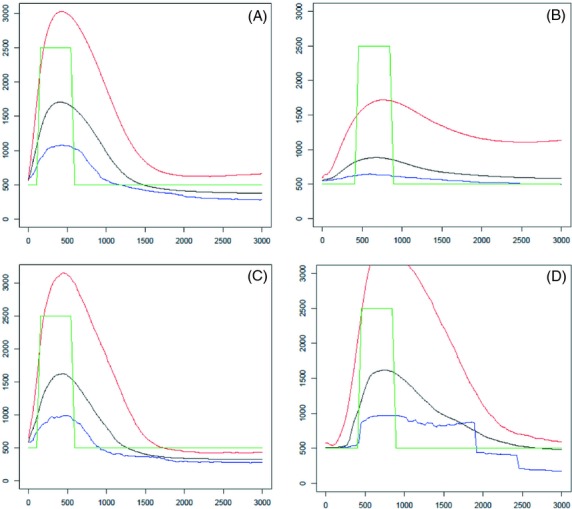
Detection of a past transient increase of population size. The simulated populations involve a fivefold increase of size from 500 to 2500 during 450 generations. The assumed mutation rate was set to 0.01 and estimations were based on 40 independent markers. Cases A and C: analysis made 100 generations after population had recovered its ancestral size (A: mean results from 100 simulations; C: results obtained from a single simulation). Cases B and D: analysis made 400 generations after population had recovered its ancestral size (B: results obtained from a single simulation; D: results obtained from a single simulation). Abscissa: time in generations. Ordinates: estimates of past population sizes from arithmetic means (red), medians (black) and modes (blue) of posterior distributions. The simulated demography is shown in green.

Fourteen demographic scenarios (described in the legends of Figs 2–4) were simulated 100 times in order to check the ability of the method to detect the effects of population expansion, of current or past bottleneck, and of a transient increase of population size in the past. For these 14 simulated cases the means and standard deviations of imbalance index (*i* = ln *θ*_2_ – ln *θ*_0_, Kimmel et al. 1998) are given in Table 3. In order to detect a change in past population size, we also calculated the ratio RN eqn (7) from present time back to some ancient time and derived its distribution over the 100 replicates. It turned out that the condition RN > 0.10 was a good indicator of a significant change of population size over the period. Table 3 gives the frequency of cases for which the ratio RN was <0.10, for several periods over which RN was calculated. Figures 4 show the mean estimates over 100 replicates of past population effective size in the different scenarios: arithmetic means, modes, and medians of posterior distribution. Figures S3–S5 show the variation of these estimates across replications.

**Table 3:** Testing past population changes. Tests were calculated on 14 series of 100 simulated populations, as described in Figs 4. Imbalance index *i*: the natural logarithm of the ratio of estimates of population size from heterozygosity and from the second moment of allele distance frequencies. RN: the ratio of the range to the mean of point estimates of population size (median of the posterior distribution) in some period, from sampling time back to the specified past time. In each case, Pr(RN < 0.10) was estimated from the distribution in the 100 simulations

Case	Reference	Imbalance index *i*	Standard deviation of *i*	Pr(RN < 0.10) (estimation)			
				Period: 0–2	0–5	0–10	0–20
Constant population size	Fig. 3A	−0.009	0.153	1.00	0.98	0.94	0.82
				Period: 0–1.2	0–2.2	0–4.5	0–9
Population expansion	Fig. 2A	−0.669	0.090	0.93	0.14	0.00	0.00
	Fig. 2B	−0.743	0.067	0.99	0.96	0.19	0.00
	Fig. 2C	−0.472	0.136	0.15	0.00	0.00	0.00
	Fig. 2D	−0.756	0.085	1.00	0.43	0.00	0.00
	Fig. 2E	−0.236	0.161	0.57	0.17	0.00	0.00
	Fig. 2F	−0.748	0.085	1.00	0.63	0.00	0.00
				Period: 0–2	0–5	0–10	0–20
Present or past bottleneck	Fig. 3B	1.204	0.214	0.14	0.00	0.00	0.00
	Fig. 3C	0.130	0.181	0.28	0.20	0.18	0.16
	Fig. 3D	−0.058	0.181	0.70	0.29	0.20	0.18
				Period: 0–5	0–12	0–25	0–50
	Fig. 3E	−0.129	0.150	0.97	0.74	0.49	0.41
	Fig. 3F	−0.082	0.161	0.96	0.86	0.74	0.71
				Period: 0–3.6	0–7.5	0–15	0–30
Transient increase in the past	Fig. 4A	−0.101	0.139	0.04	0.03	0.01	0.00
	Fig. 4B	0.082	0.156	0.49	0.31	0.21	0.18

##### Posterior distribution of past effective size

**Figure 5:**
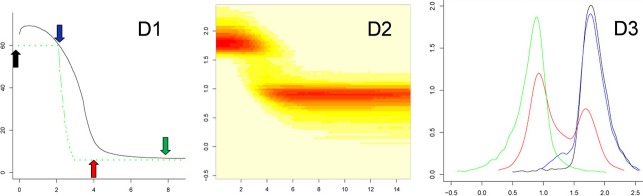
Posterior distribution of effective population size in the past after exponential expansion. The Figure provides detailed results for one of the 100 simulated cases of Fig. 2D. (D1) median estimates; (D2) two-dimensional joint distribution of past time and past effective size; (D3) detailed posterior distributions of population size at the present time and at three times in the past. Abscissa: (D1 and D2) reduced past time; (D3) decimal logarithms of population size (theta units). Ordinates: (D1) median of the posterior size distribution (in black, theta units), the simulated demography is shown in green; (D2) decimal logarithm of population size (theta units); (D3) densities of the posterior distribution of the logarithm of population size (theta units): at the present time (black), and at reduced times 2 (blue), 4 (red), and 8 (green) in the past. Colored arrows in box D1 indicate the times when the distributions were calculated.

**Figure 6:**
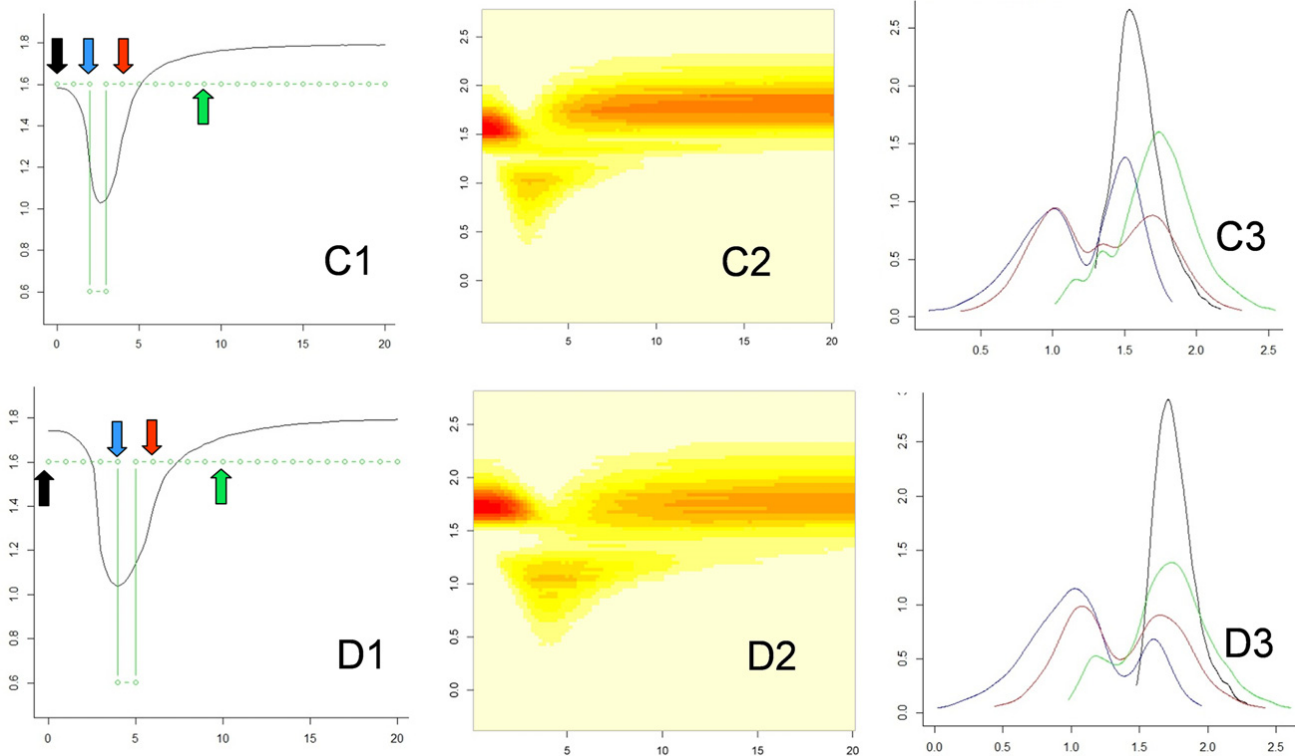
Posterior distribution of effective population size in the past after a bottleneck. The Figure provides detailed results for one of the 100 simulated cases of Fig. 3. (C1–C3) Outlines of the posterior distributions, as in Fig. 5, for a sample taken 200 generations after the bottleneck; (D1–D3) outlines of the posterior distributions, as in Fig. 5, for a sample taken 400 generations after the bottleneck. Abscissa: (C1, C2, D1, and D2) past time in reduced time; (C3 and D3) decimal logarithms of reduced population size (theta's). Ordinates: (C1 and D1) median of the posterior distribution of the logarithm of population size [log(theta), in black], the simulated demography is shown in green; (C2 and D2) decimal logarithm of population size log(theta); (C3 and D3) densities of the posterior distribution of the logarithm of population size (theta units) at the present time (black), and in the past: at times 2 (blue), 4 (red), and 8 (green) for (C3) and at times 4 (blue), 6 (red) and 10 (green) for (D3). Colored arrows in C1 and D1 indicate the times when the distributions were calculated.

We report also results concerning single simulations to illustrate the ability of the method to get a detailed view of posterior distributions. Figures 5 and 6, using the additional functions included in the package, illustrate these possibilities. For a number of cases of expansion, bottleneck and transient increase, *MSVAR* was run on the same data. Table 2 shows the comparison of results obtained from both methods.

##### Migration scheme

**Figure 7:**
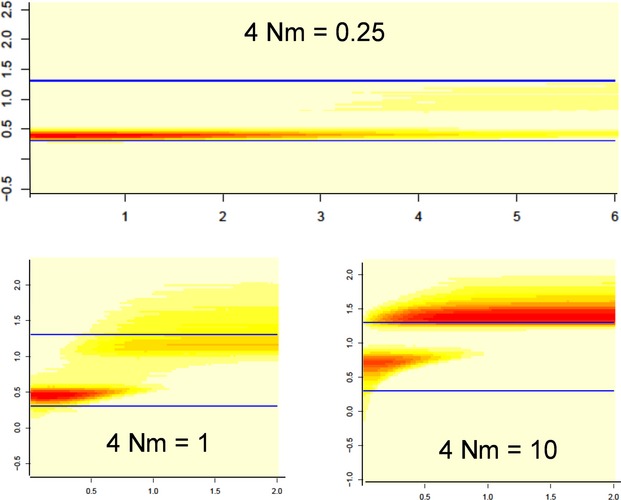
Effects of migrations on effective population size estimation. The posterior distribution of past effective size estimated from samples within a small population (*θ* = 2) submitted to immigration from a larger population (*θ* = 20), for three rates of immigration (4*Nm* = 0.25, 1, and 10). Ordinates: logarithm of population size (theta units); the blue lines correspond to the actual sizes of the small and the large populations. Abscissa: time in the past (reduced time scale).

Figure 7 illustrates the application of the method in the case of a population submitted to permanent immigration from a larger population, according to the model outlined in Data S2. Results show how the population sizes of the sampled population or of the external population were recovered depending on the 4*Nm* parameter and on the time in the past.

### Atlantic salmon data sets

**Figure 8:**
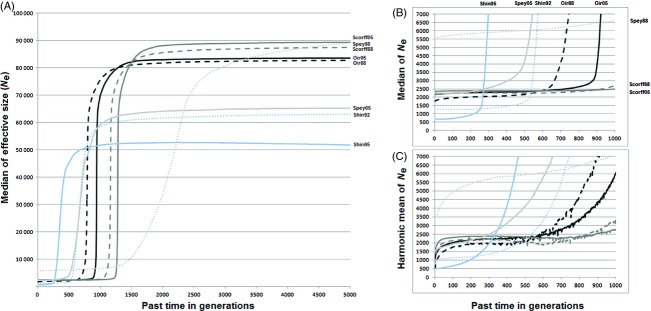
Estimations of Atlantic salmon's effective population sizes from Oir, Scorff, Shin, and Spey rivers at two sample dates (1988 or 1992 and 2005). (A) Estimates of population sizes from the posterior medians, from year 2005 to 5000 generations ago. (B) Zoom on the last 1000 generations (posterior medians). (C) Zoom on the last 1000 generations (posterior harmonic means). Each line is associated to a population: Oir 2005 (Oir05) black line, Oir 1988 (Oir88) black dashed line; Scorff 2005 (Scorff05) in dark gray line, Scorff 1988 (Scorff88) in dark gray dashed line; Shin 2005 (Shin05) in light blue line, Shin 1992 (Shin92) in light blue dashed line; Spey 2005 (Spey05) in light gray line, and Spey 1988 (Spey88) in light gray dashed line.

The method was used to estimate the current and past effective sizes of wild Atlantic salmon populations sampled in two countries, France (Oir and Scorff) and Scotland (Shin and Spey) in 1988, 1992 and 2005 (Nikolic et al. 2009a). Estimations of sizes were searched for from sampling time to 5000 generation ago. The results revealed a large past ancestral size (median around 50 000–90 000) and a lower current size (Fig. 8, Table 4), assuming a mutation rate of 0.0003 (Nikolic et al. 2009b). The general patterns of effective size (Fig. 8) were similar for the four populations, except for the 1988 sample of river Spey. Deriving the posterior distribution of the Time to the Most Recent Common Ancestor (*T*_MRCA_) of a pair of alleles from the distribution of effective size (eqn A2 in Data S1) confirmed that these populations underwent a bottleneck around 300–1500 generations ago. According to Fig. 8A, the times (*g*_*b*_ generations ago) of the bottleneck were estimated, for Oir 2005 (*g*_*b*_ = 900), Oir 1988 (*g*_*b*_ = 700), Scorff 2005 (*g*_*b*_ = 1300), Scorff 1988 (*g*_*b*_ = 1000), Shin 2005 (*g*_*b*_ = 300), Shin 1992 (*g*_*b*_ = 500), Spey 2005 (*g*_*b*_ = 500) and for Spey 1988 (*g*_*b*_ = 1500).

**Table 4:** Distribution of Oir, Scorff, Shin and Spey Salmon population sizes. Arithmetic mean, harmonic mean, mode, median, and quantiles 5% and 95% (q5%, q95%) of present (*N*_0_) and past (*N*_*a*_) (5000 generations ago) effective sizes for Oir, Scorff, Shin and Spey Atlantic salmon populations at the two sampling dates (2005 and 1988 or 1992). The census size (*N*) of populations are also shown and the ratio *N*/*N*_0_ of *N* to the harmonic mean (*H*) and median (*M*) of *N*_0_

	French populations								Scottish populations							
		
River/sample	Oir 2005		Oir 1988		Scorff 2005		Scorff 1988		Shin 2005		Shin 1992		Spey 2005		Spey 1988	
Census size (*N*)	130		260		1000		NA		3000		3000		<60 000		<60 000	
Effective size	*N*_0_	*N*_*a*_	*N*_0_	*N*_*a*_	*N*_0_	*N*_*a*_	*N*_0_	*N*_*a*_	*N*_0_	*N*_*a*_	*N*_0_	*N*_*a*_	*N*_0_	*N*_*a*_	*N*_0_	*N*_*a*_
Arithmetic mean	2200	87 000	1800	86 000	2400	95 000	2400	93 000	2000	70 000	3400	90 000	4700	85 000	8300	110 000
Harmonic mean	1100	83 000	480	81 000	1400	87 000	850	85 000	480	52 000	960	61 000	1500	64 000	3300	64 000
Mode	2300	79 000	1900	79 000	2400	85 000	2100	82 000	620	44 000	1300	61 000	2100	65 000	5700	86 000
Median	2200	84 000	1800	83 000	2400	89 000	2100	87 000	680	52 000	1300	63 000	2300	65 000	5500	89 000
q5%	510	66 000	130	63 000	1000	67 000	360	67 000	180	37 000	440	38 000	570	44 000	1200	21 000
q95%	3200	120 000	3800	120 000	3100	140 000	4500	140 000	7700	150 000	11000	230 000	17000	180 000	20000	240 000
*N*/*N*_0_ (*H*)	0.12		0.54		0.70		NA		6.3		3.1		<40		<18	
*N*/*N*_0_ (*M*)	0.06		0.15		0.43		NA		4.4		2.3		<27		<11	

## Discussion

### The estimation method

#### Technical efficiency

A first observation is that the present method behaves as well as the best known estimates in the case of constant population size, getting the same dependence of precision on marker diversity and on the number of markers. For a constant population size, estimates given by *θ*_0_ based on current heterozygosity and by the mode of the posterior distribution turned out to be the best ones (Fig. 1). Except for a low variability (*θ =* 1), the harmonic mean and the median showed similar efficiency, while the arithmetic mean was generally less efficient and more sensible to a reduction of the number of markers.

The method is based on an approximation of the likelihood of the mean values of allele distance frequencies by a multivariate normal. Table 1 shows how the value of this approximation depends on the number of markers. It must also be stressed that the approximation becomes weaker if markers are less variable (lower theta values). Increasing the number of markers would then be necessary but not always sufficient. The approximation may become quite weak if the number of markers is low or if some frequencies are very low. Deriving the distribution of means over markers of allele distance frequencies assumes that all the markers follow the same mutational process. It is often difficult to consider that differences between allele frequencies profiles are due to different mutational processes, because the variation expected from drift eqn (9) is extremely large. Applying some normalization between markers to make the frequency profiles more regular could in fact hide the natural variations and bias inferences. However, markers may be less regular than in the simulations, and checking the dependency of results on the choice of markers may be required. If hundreds of markers are available, as for human populations (Rosenberg et al. 2002; Tishkoff et al. 2009), one could sample subsets of about 50 markers and check the stability of results across sets of markers. This would be approximately equivalent to the repeated scenarios considered above and could allow the times of important size change to be detected from the variation over repetitions of size estimates. Compared to approaches aimed at considering the full exact likelihood of data, either through an MCMC approach (Wilson and Balding 1998; Beaumont 1999; Wu and Drummond 2011) for microsatellite markers or through an analytical derivation of likelihood for small samples (Lohse et al. 2011, for the infinite-sites model) the present approach is based on simplifications and does not take account of variable mutation rates. However, it allows the effects of priors and of the mutation model on past population sizes estimation to be easily tested, because calculations are very fast permitting alternative models to be compared.

#### Detection efficiency of past population size

The most interesting feature of the method is its ability to recover the dynamics of size variations and to detect transient changes, not only general tendencies such as monotone growth or decline of population size. Under changing population size, the median of the posterior distribution was found to be the most robust estimator. The harmonic and the arithmetic means were sensible to extreme small and large values that are generated by the simulation, for recent and ancient times, respectively. The occurrence of bimodal posterior distribution of effective size makes estimating size through the mode sensible to a shift between local modes. As a consequence, the times when population size has undergone a change may be strongly biased when using the mode. The present method seems quite more useful than a single criterion like an imbalance index (Table 3) that does not seem able to detect transient changes, except in very sharp situations. The strong current bottleneck (Fig. 3B) and past important expansions (Fig. 2B,D,F) resulted in highly significant imbalance indices and were well detected. On the contrary, except for slow growth (Fig. 2A), using imbalance indices did not allow the currently growing populations (Fig. 2C,E), or the transient past increase of population size (Fig. 4) to be detected. In contrast the present method provided good evidence of these events, for example using the RN ratio (eqn 7, Table 3) and deciding that population size has changed if RN > 0.10. For expansion cases (Fig. 2), this test was always very significant provided the period of time used to calculate RN is long enough. For past bottlenecks, the method seemed very efficient when population size remained small after the bottleneck (Fig. 3B), would miss detection with a probability of about 0.20 for relatively ancient bottleneck (Fig. 2C,D), and lack efficiency for quite ancient events (Fig. 2E,F). In the case of a transient past expansion (Fig. 4A,B), a quite good efficiency was also observed. It may also be noted that considering several periods to calculate RN, may allow the time when a transient change occurred to be evaluated, although these times seemed often overestimated. In the case of a constant population size, the observed values of Pr(RN < 0.10) from 0.82 to 1.0 are estimates of the power of the test.

Provided past events of population size changes are not too ancient and strong enough the method provided a good view on past demography. However, it was observed that under current expansion, the present size could not be recovered (Fig. 2A,C,E). The signal of expansion was clear but the current population size was underestimated, the larger the expansion rate the larger the bias. Estimates of effective sizes in the recent past suggested that population had reached a plateau. In these cases, using *MSVAR* provided more valuable estimates of present population size (Table 2). If the analysis was carried out after the population reached a new plateau (cases B, D, and F where a delay between 2 and 3 in the reduced time scale was applied), *VarEff* provided a correct picture of the actual history, although the time when expansion occurred seemed overestimated. The different rates of expansion assumed in the simulations led to similar profiles of estimated sizes, with low differentiation between expansion rates. In all the cases, the ancient small population size was correctly estimated, especially when using the median estimate. Conversely, *MSVAR* failed in these cases to provide correct estimates. Large overestimations of the current size and of the time since expansion were obtained, while ancestral population sizes were underestimated (Table 2). The increase of uncertainty for the mode and the median *VarEff* estimates corresponded to the times when population size had undergone its expansion (Figure S3). This burst of uncertainty was observed in the period when the population underwent demographic change, hence was lasting longer for slow expansion (Figure S3B) than for fast expansion (Figure S3F). The precision on the current size was roughly equal to that given by the *θ*_2_ estimate for constant sizes (Fig. 1), while precision on the ancestral size was about 50% lower.

Detecting past bottlenecks relied on the chance of observing pairs of alleles that had coalesced during it. In the cases of Fig. 3, this corresponded to pairs of alleles whose *T*_MRCA_ was between *G*_1_, the time when population recovered its ancestral size *N*_0_, and *G*_2_, the time when the size was reduced to *N*_1_ (*N*_0_ = 1000, *N*_1_ = 100, *G*_1_ = 200 and *G*_2_ = 300 in Fig. 3C). The probability that *T*_MRCA_ is less than *G*_1_ is equal to the inbreeding index 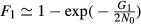. The probability that *T*_MRCA_ is between *G*_1_ and *G*_2_ is then equal to (1−*F*_1_) times the probability that coalescence occurred before *G*_2_−*G*_1_ generations in the population of size *N*_1_, i.e., 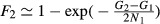. The probabilities that a pair of alleles has coalesced after, during and before the bottleneck are therefore, respectively, *F*_1_, (1−*F*_1_) *F*_2_, and (1−*F*_1_) (1−*F*_2_). These values are approximately (0.10, 0.36, 0.54), (0.18, 0.32, 0.50), (0.63, 0.15, 0.22) and (0.86, 0.06, 0.08) for Fig. 3C, D, E, and F, respectively. Detecting the bottleneck depends on the chance to sample a sufficient number of pairs of alleles corresponding to the second class: the bottleneck must be strong enough (large *F*_2_ value) and not too old (small or intermediate *F*_1_). For the same reason, accessing the ancient population size, before the bottleneck event, is possible only if it is not too ancient. Also, it must not be too strong: for a large *F*_2_ value the number of alleles derived from the ancestral population is greatly reduced, to about 2/*F*_2_ – 1 ≃ 4 in Fig. 3 (Chevalet 2000). This may explain why the ancestral population size seemed underestimated even for a quite ancient bottleneck (Fig. 3E). Using *MSVAR* in these cases suggested a very recent and very fast increase of population size from an ancestral smaller population size, indicating for example the doubling of population size in the last two generations, for a recent bottleneck (Table 2, Fig. 6C). When the bottleneck is very ancient (Fig. 3F), no signal could be detected, which is expected since a new mutation-drift equilibrium was recovered. The precision on the current population size remained correct, while it was roughly halved for the ancestral population size (Figure S4). In case B in which the population was observed during the bottleneck, a large increase of uncertainty arose for the period when the population underwent its change.

Transient increases of population sizes were also detected, although the transient size seemed underestimated, with a sharper underestimation when analyses were performed a long time after the population had recovered its ancestral small size (Fig. 4). Detecting an increase of population size from allelic diversity requires that novel diversity be detected, i.e., that new alleles reached measurable frequencies. A very short period with a large population size would just result in allele frequencies being unchanged, so that no signal could be observed when looking at genetic diversity. If the population size is rapidly set from *N*_0_ to a new larger *N*_1_ value, new mutations can accumulate to eventually reach new mutation-drift equilibrium. A rough quantitative evaluation of such effects can be derived using the Infinite Allele Model for which diversity is characterized by the frequency of heterozygotes. Before the increase (time *G*_2_), the equilibrium heterozygosity was *H*_0_ = 4*N*_0_*μ*/(1 + 4*N*_0_*μ*). From this time *G*_2_ to the time *G*_1_ when the population recovered its ancestral size *N*_0_, the expected heterozygosity raised from *H*_0_ to 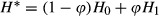, where *H*_1_ = 4*N*_1_*μ*/(1 + 4*N*_1_*μ*) is the equilibrium heterozygosity with size *N*_1_ and where 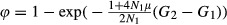 is a measure of the approach to the mutation-drift equilibrium at size *N*_1_. Detecting the event requires that the increase H*−*H*_0_ be significant, hence that both *H*_1_−*H*_0_ and *φ* be large enough. In addition, the time *G*_1_ must also be large enough, so that a sufficient proportion of coalescence events involve new alleles generated during the burst of population size. The lack of time needed to let new mutations spread in the population may also explain the inability of the method to provide reliable estimates of the current size of a population in fast exponential expansion (Fig. 2A,C,E). In these cases, allele frequencies remain almost unchanged and estimations rely on the frequencies achieved some time ago. The recovery of the ancient size seemed correctly estimated, but as observed for expansion scenarios (Fig. 2), the past time when population size began its expansion seemed overestimated, with a larger error when estimation was carried out later (case B versus A, and D versus B). In the cases shown in Fig. 4B,D, using *MSVAR* provided valuable estimates of present population size, and suggested that population underwent a decrease from a larger ancestral population size. As in the cases of past bottlenecks, transient past sizes are ‘hidden’ to the *MSVAR* approach that strongly relies on the assumption of a monotone size evolution, and *MSVAR* suggested then very fast population change. For *VarEff*, the precision on present size remained good, while it was lowered for ancestral sizes, as observed in the case of a bottleneck (Figure S5).

#### Sensitivity to gene flow

The population model refers to a single isolated population. If the population is in contact with an external gene pool, coalescence events between alleles may happen outside the population. The method is based on eqn (A4) (Data S2), hence it returns estimates of a function *N*(*t*) meant as the past size of an isolated population from which alleles were sampled. When data are not drawn from an isolated population, the estimated function *N*(*t*) should be re-interpreted. For the simple migration model illustrated in Fig. 7, analytical calculations (Data S2) show that the distribution of coalescence time depends on two parameters: 4*Nm* (where *m* is the proportion of immigrant gametes per generation) and the ratio *ε* of the considered population size *N* to the size of the larger external population. Figure 7 shows what happened when applying the present method to such data, considering several values of the migration rate: when 4*Nm* is small, the estimated population size remained in the order of magnitude of the true *N* while increasing with *m*. For larger values, the studied population seemed to ‘vanish’ some time ago, the larger the 4*Nm* value, the most recent this artifactual event. Looking at the complete posterior distribution indicated a bimodal distribution with a second local mode corresponding to the size of the large external population. For large 4*Nm* values, results were very close to those expected for a sample drawn in the external large population. According to eqn (8), the predicted times of these apparent past changes of effective size are 3.1, 1.5, and 0.22 for the three cases, respectively, which is in good agreement with Fig. 7. This analysis provides an explanation for the wrong detection of strong bottleneck by a program like *MSVAR* when analyzing data generated according to an island model with constant population sizes, and we could propose an estimation for the time when a false bottleneck it detected eqn (8). Like *MSVAR*, our method is based on estimated distributions of coalescence times of alleles. In the case of immigration, similar alleles (at low *k* distance) drawn from the population derive mostly from an allele within the population, i.e., during the ‘scattering phase’ (Wakeley 1999). Distant alleles are likely to have coalesced in the large external population (the ‘collecting phase’), as soon as one of them was an immigrant. This leads to a bimodal distribution of coalescence times between two alleles, and hence a bimodal distribution of past population size when coalescence times are turned into population sizes. As pointed out in several works (Nielsen and Wakeley 2001; Ptak and Przeworski 2002; Nielsen and Beaumont 2009; Chikhi et al. 2010; Peter et al. 2010), about the same distribution of coalescence time holds for alleles drawn in a population after a bottleneck. If these alleles did not coalesce since the beginning of the bottleneck, their coalescence has occurred when population size was larger. Although both kinds of events (coalescence during or before the bottleneck) do not occur in the same periods of time, the variability across markers may cause overlaps in the predicted time of the bottleneck and lead to bimodal population size distributions as shown for example in Fig. 6 in a case of bottleneck.

#### Sensitivity to mutation models

Detecting past variations of effective size is also sensible to the assumed mutation model. For example, if markers follow a geometrical model of mutation (Whittaker et al. 2003; Watkins 2006), the expected value of the imbalance index is increased, suggesting a past bottleneck: with a *c* parameter value of 0.5 [corresponding to a mean value 1/(1−*c*) = 2 of the number *r* of steps for each mutation event] imbalance indices are 1.55, 1.22, 1.01 and 0.89 for a constant population size *θ* equal to 1, 4, 8, and 12, respectively. A general approach to the question was proposed by Wu and Drummond (2011) who considered more realistic mutation models and could take account of marker specific mutation processes. Although less general, the much faster *VarEff* method allows various mutation models to be considered and some trials were performed to check its sensitivity. For constant population size, using data simulated under the Single Step Model (SSM) but assuming a more complicated model to perform estimation (allowing for a Two Phase Mutation model or for a Geometrical model), the current population size was underestimated and ancient size estimate was even smaller, suggesting past expansion. Conversely, assuming SSM in the analysis of data that were generated under a more complicated mutation model led to an overestimation of ancient population size and suggested the false detection of a current bottleneck, as predicted by the behavior of imbalance indices. A look at the fit of the model to data (the mean value of the quadratic departure of observations from the model, eqn (5)) allowed in general the right model to be identified as that with the best fit. Simulating bottlenecks led to similar biases for ancient population sizes, but estimates of current population sizes were only weakly sensitive to the assumed mutation model. This may be understood since detection of current bottleneck relies mostly on the variation of allele frequencies in the last generations. It depends more on drift than on mutations, while inference on ancient frequency distributions is strongly dependent on the mutational process. The impact of using a different model for data generation and analysis remained qualitative. From a practical point of view, it may be suggested to run the estimation using several mutation models, check the fitness of models to data, and test the robustness of results across equally fitted models. The analysis was performed for the salmon populations and suggested that SSM provided the best fit in most cases. However, for some populations the Two Phase Model or the Geometrical model, with a small *c* value of 0.2, gave a slightly better fit, suggesting that the ancestral population sizes given in Table 4 and Fig. 8 might be slightly overestimated.

### The history of Atlantic salmon populations

During the last 30 years, the decline of wild salmon on both sides of the North Atlantic (Parrish et al. 1998; Jonsson and Jonsson 2004) has affected populations to differing degrees (Hawkins 2000). Owing to their homing behavior, salmonids are an ideal species for assessing the influence of population structure on *N*_*e*_ estimations. The *VarEff* model was applied on European wild Atlantic salmon populations for which the genetic diversity and structure have been previously studied (Nikolic et al. 2009a). The four populations studied are pressured by different factors and are therefore subject to varying conservation and management strategies. Because their characteristics are well understood (Baglinière and Champigneulle 1986; Butler 2004; Baglinière et al. 2005; Butler et al. 2008) and they have large differences in abundance, they provide a useful opportunity to evaluate tools for estimating *N*_*e*_.

The results obtained by *VarEff* (Table 4) are consistent with other coalescent models with estimates of the current and ancestral effective sizes nearer to those given by *MSVAR* than to those given by *DIYABC* (Nikolic et al. 2009a). The shape of effective sizes' fluctuations from sampling to ancestral times revealed a homogeneous history for the Atlantic salmon populations from France and Scotland with a recent bottleneck. The Oir and Scorff older samples (1988) had lower effective sizes than recent samples (2005). On the contrary, Shin and Spey older samples had higher effective sizes than recent samples. The populations were subject to a global decrease in wild salmon stocks coming from a common larger ancestor population (around 50 000–90 000 effective individuals) dating back to the last glaciations. Regarding the median estimates, a bottleneck is suggested about 300–1500 generations ago.

Comparison of census sizes to estimated current effective sizes showed sharp differences between populations (Table 4). Using the harmonic mean to estimate effective sizes, the disparity increased from the past samples to the current samples. From 1988 to 2005, Oir population showed an increase of current effective sizes while census sizes were decreasing. Spey and Shin populations have a current effective size lower than census size from past (1988 and 1992) to 2005. The ratios (*N/N*_0_) of census size (*N*) to effective size at sample time (*N*_0_) were <1 for the French populations and larger than 1 for the Scottish populations suggesting a better status for the Scottish populations. Considering the harmonic mean values of effective size, the French populations seem more impacted than the Scottish ones and a very recent decrease was revealed in the last generations. According to the harmonic mean estimates, these decreases occurred these last five decades (since 1950) and were detected in both samples from the four populations. They were of about 30–50% for Oir, 30–45% for Scorff, 9–10% for Shin, and 4–6% for Spey, revealing a much higher drop in France than in Scotland.

The observed ancient bottleneck which might date back to the last glaciations could also be interpreted under an immigration model, according to which the different populations could be impacted by recurrent immigration from a common large metapopulation. Under this model eqn (8), estimates of current (at sampling time) and past effective sizes (Table 4) and of the times when the ancient bottleneck is detected (Fig. 8) allow the order of magnitude of immigration rates *m* to be derived. Suggested values of *m* are, respectively, about 0.0022 and 0.0014 for Oir and Scorff rivers for both samples. On the contrary, Scottish rivers give contrasted results, with a value of about 0.0034 for the 2005 Spey and 1992 Shin samples, but a quite lower value of 0.0006 for the 1988 Spey sample, and an increased rate of 0.007 for the 2005 Shin sample.

Gene flow may reduce the differentiation between populations and conversely the resident forms may increase their differentiation because the sea acts as a barrier to dispersal. The homing behavior of Salmon (Skaala and Naevdal 1989; Debowski and Bartel 1995) prevents important gene flow so that the species tends to be structured into genetically distinct populations of a geographical area to another or a watershed to another, indicating the possibility of local adaptation. Straying, leading to the contribution to reproduction in subsequent generations and promoting gene flow remains limited. Generally, it is considered that the homing is very strong in Atlantic salmon and the percentage of strayers varies between 2% and 6% (Stabell 1984; Quinn 1993; Altukhov et al. 2000; Jonsson et al. 2003). Natural gene flow may not be a real problem in salmon but the artificial gene flow must be one modifying the evolution of coalescent effective size. An introduction of non-native unknown Scottish juveniles into Scorff shifted the bottleneck and led to an artificial increase of the effective size, as demonstrated in our simulations to test the effects of migrations on effective population size estimation (Fig. 7). Oir population is in the same case with the introduction of juveniles since the 1990s from the Sélune River considered as the pool source watershed. In the Shin river, the long-term artificial stock enhancement program using fish of native origin has been set up to mitigate the blocking of freshwater habitat by hydroelectric dams in the 1950s and have reduced genetic variability of Shin population, which led to underestimate current effective size. The same holds for the Spey population, where fish of native origin were used since the 1970s. Analyzing the two Spey samples also showed strong differences between their demographic histories (Table 4, Fig. 8), corresponding to temporal genetic differentiation. The 2005 Spey sample was taken from the upper catchment, where spring running fish are known to originate (Laughton 1991). The 1988 samples came from several months, mainly July, August and September, while 2005 samples came from 1 month. Large populations in rivers such as the Spey are known to contain genetically distinct population units (sub-stocks), which differ in the timing of their return migration (Stewart et al. 2002; Jordan et al. 2005). The Spey 1988 sample could represent several sub-stock of spring running fish while the 2005 sample would correspond to one stock, explaining the higher effective size of the past Spey population. Since the gain or loss of variability is used to trace the history of a population, any forces that were not included in this model must be considered to recover a correct interpretation of results.

Generally, the most important factor reducing the effective size and genetic variability are fluctuating population size in different generations, followed by variation in family size, variation of mating system (i.e., polygynous versus polyandrous), and variation in sex-ratio of breeding individuals (Frankham 1995; Hedrick and Kalinowski 2000). Nikolic et al. (2009a) tried to explain the high genetic variability observed in populations with a small census size (Oir and Scorff) by two hypotheses: (i) these populations underwent a very recent bottleneck 25–100 generations ago and (ii) the high proportions of eggs fertilized by parr in Oir and Scorff (Baglinière and Maisse 1985; Baglinière et al. 1993) make precocious mature parr contribute significantly to the genetic variability, as reported in almonds by Garcia-Vazquez et al. (2001) and by Johns and Hutchings (2001, 2002). According to the present study, the most drastic bottleneck underwent by the wild Atlantic salmon occurred hundreds of generations ago and was followed by another decrease these last decades in France, which favors the second hypothesis. The precocious mature parr contribute to enlarge the effective population size, which may explain the higher effective size at the sample time *N*_0_ in the French populations (Oir and Scorff) compared to their census size *N* (Table 4). Regarding this disparity in Oir, between current effective (*N*_0_) and census (*N*) size changes from 1988 to 2005 (increased effective size versus lowered census size), an increase of precocious mature parr is suggested in the Oir population. The precocious mature parr seem highly active in the French populations probably to balance the negative anthropic impacts. These new biological strategies may have to be measured within different watersheds in France to list the different degrees of establishment. This process could increase the genetic differentiation between populations from a watershed to another, indicating the possibility of local adaptation but also reducing the gene pool of the species.

## Conclusion

As the catastrophic loss of biodiversity continues unabated, guidelines for how extinction risk is related to population size *N*_*e*_ should be a high priority in conservation biology (Shaffer et al. 2000; Hare et al. 2011). For evolutionary matters, the effective population size is a prime concern. Therefore, there is a need to develop efficient methods aimed at detecting past variations of effective population size. Here, we have proposed a new fast method (*VarEff*) based on microsatellite data, which remain valuable markers to assess genetic diversity in natural populations, for which complete genome sequence analysis is not yet available on a large scale. Due to their high mutation rate, microsatellites allow recent history to be investigated and provide complementary views. The *VarEff* model relies on an approximation of the likelihood of data from which a fast algorithm allows size variations to be efficiently detected, without any prior hypothesis about the demographic history such as monotone growth or decline. The approximation relies on the strong hypothesis that markers share the same mutation process and the same mutation rate. This limit could be overcome considering several sets of dozens of markers, provided many ones (hundreds) are available, and testing the robustness of results. Among its advantages over methods like *MSVAR* (Beaumont 1999), it was found that results did not depend much on priors, and that their dependence on the assumed mutation model could be explored. Trials with various hypotheses allow the robustness of qualitative results to be checked, and the fitness of alternative models may help choose the best model. However, a deeper analysis of the influence of mutation models on results might be worth further works, following for example the analysis of Wu and Drummond (2011) even if their approach is very time consuming. We have given some insight into some sources of erroneous conclusion about recent changes of effective population size. For example, we showed how choosing an inappropriate mutation model, or ignoring the effects of gene flow may mimic a bottleneck. Presently, the method allows mutation models to be compared, and extensions to evolutionary schemes involving migration between several populations could be developed, providing an alternative efficient approach to those based on ABC (DIYABC, Cornuet et al. 2008).

## Data archiving statement

The *VarEff* software and data examples are available at http://cran.r-project.org/web/packages/VarEff.

## Supporting Information

**Figure S1.** Accuracy of population size estimates from the Mode and the Median as function of the diagonal *λ* parameter.

**Figure S2.** Accuracy of the estimation of the current population size from estimates in the past.

**Figure S3.** Coefficients of variation of effective population size estimates after exponential expansion.

**Figure S4.** Coefficients of variation of effective population size estimates after a bottleneck.

**Figure S5.** Coefficients of variation of effective population size estimates after a transient increase.

**Data S1.** Genetic diversity at microsatellite markers in a finite population. **Data S2.** The distribution of the *T*_MRCA_ in a simple migration model.
