# Supplementary figures and images for: Detecting past changes of effective population size
Source: Evol Appl. 2014 Jun 16;7(6):663–81. doi: 10.1111/eva.12170 (PMC4105917; doi:10.1111/eva.12170)

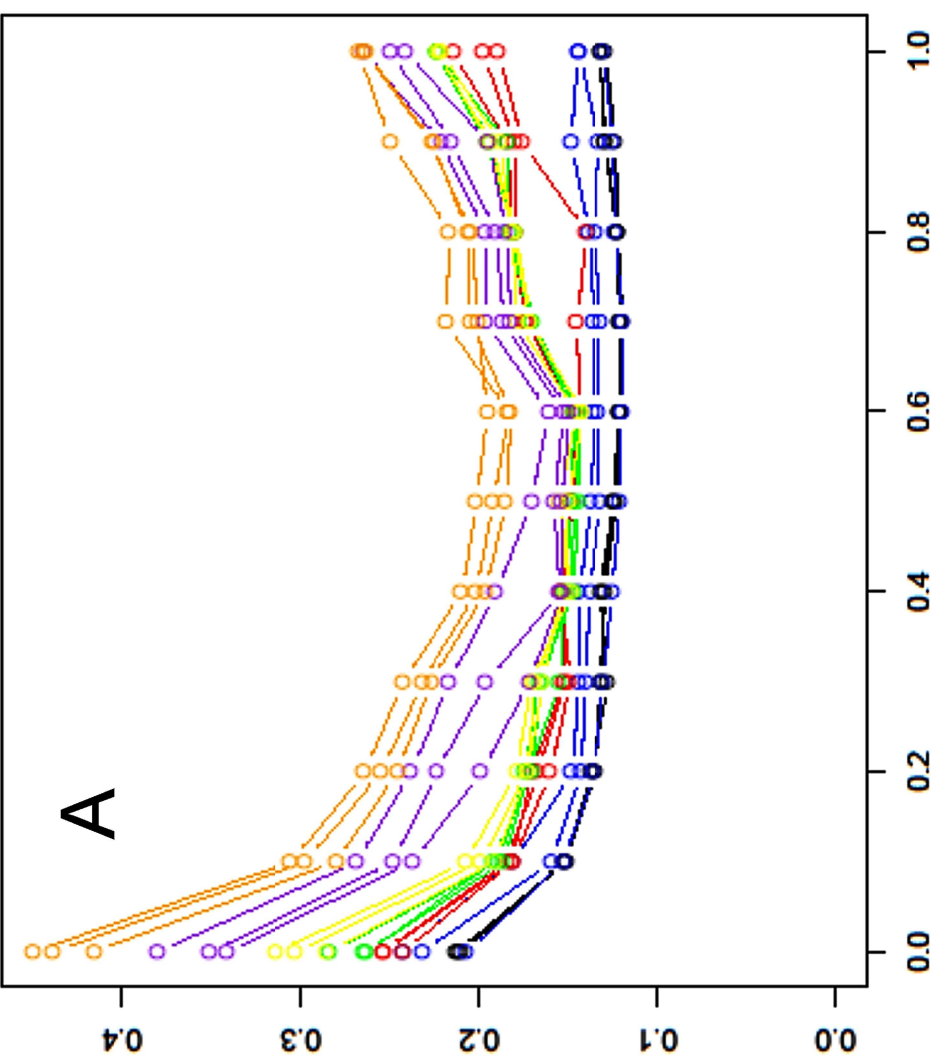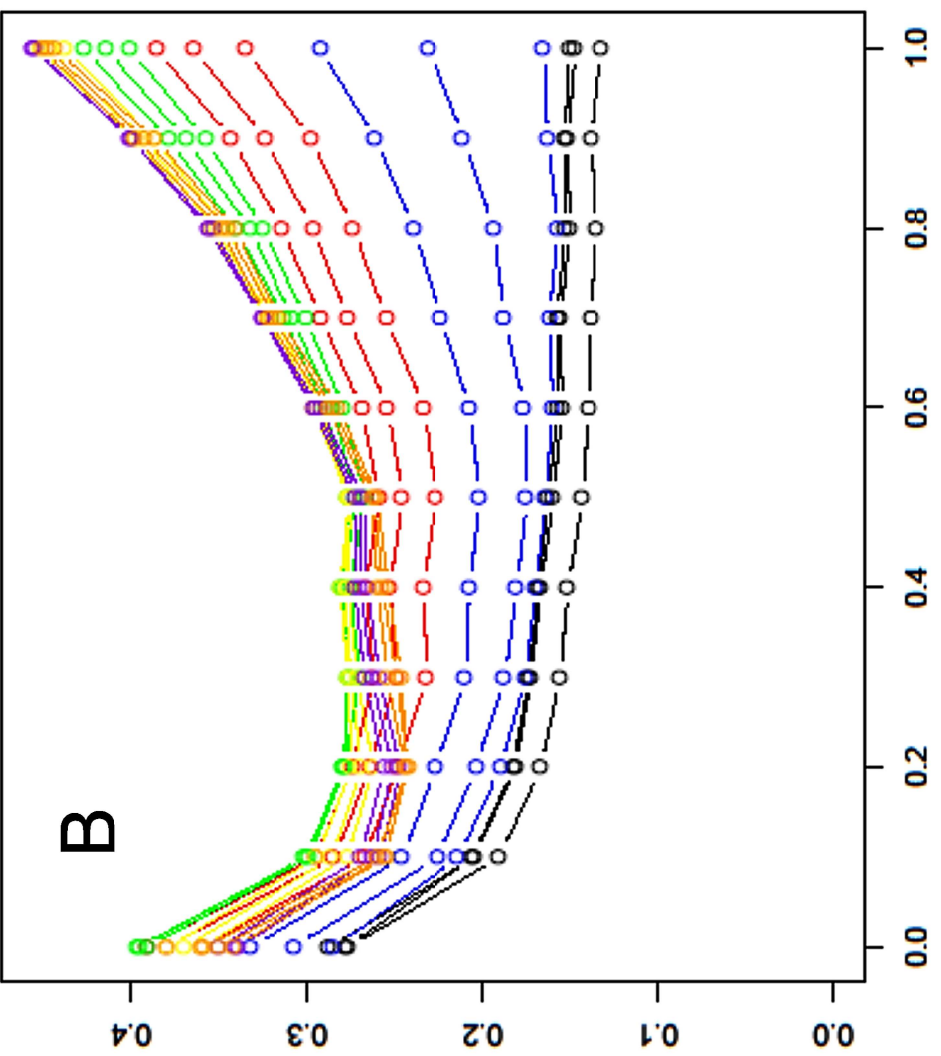

Supplement: Supplementary file 1 — Figure S1. Accuracy of population size estimates from the Mode and the Median as function of the diagonal λ parameter. [file eva0007-0663-SD1.pdf]

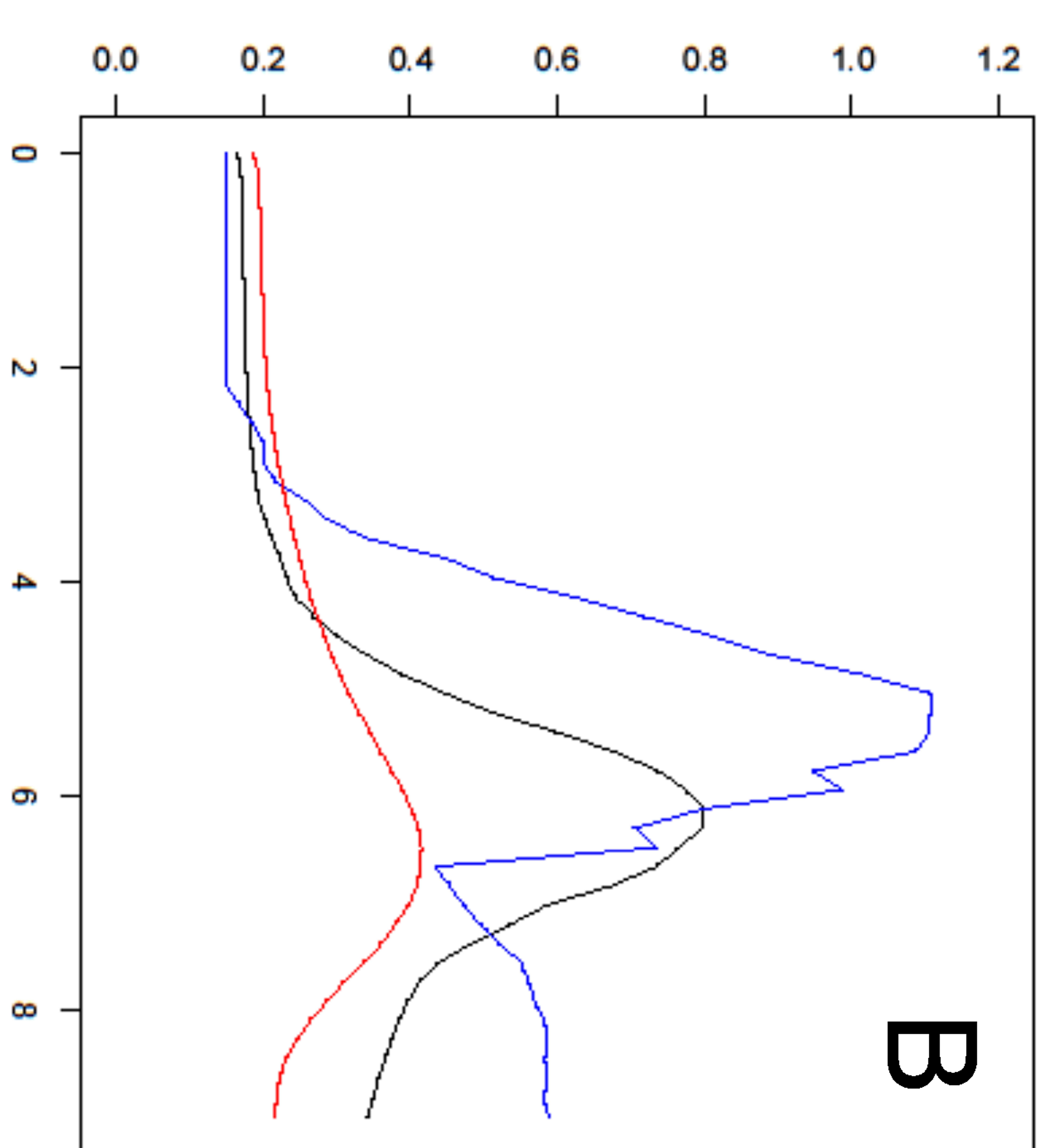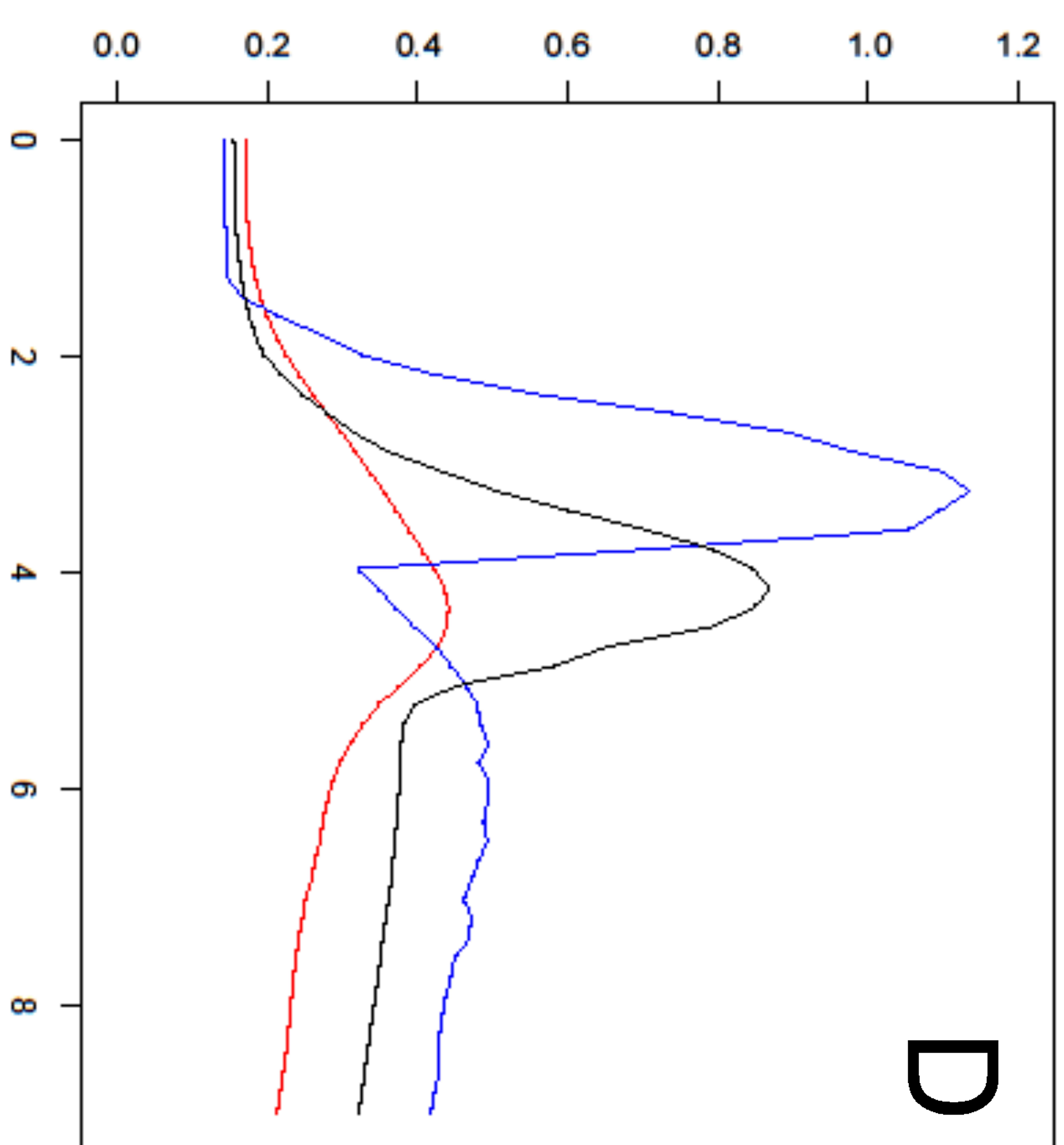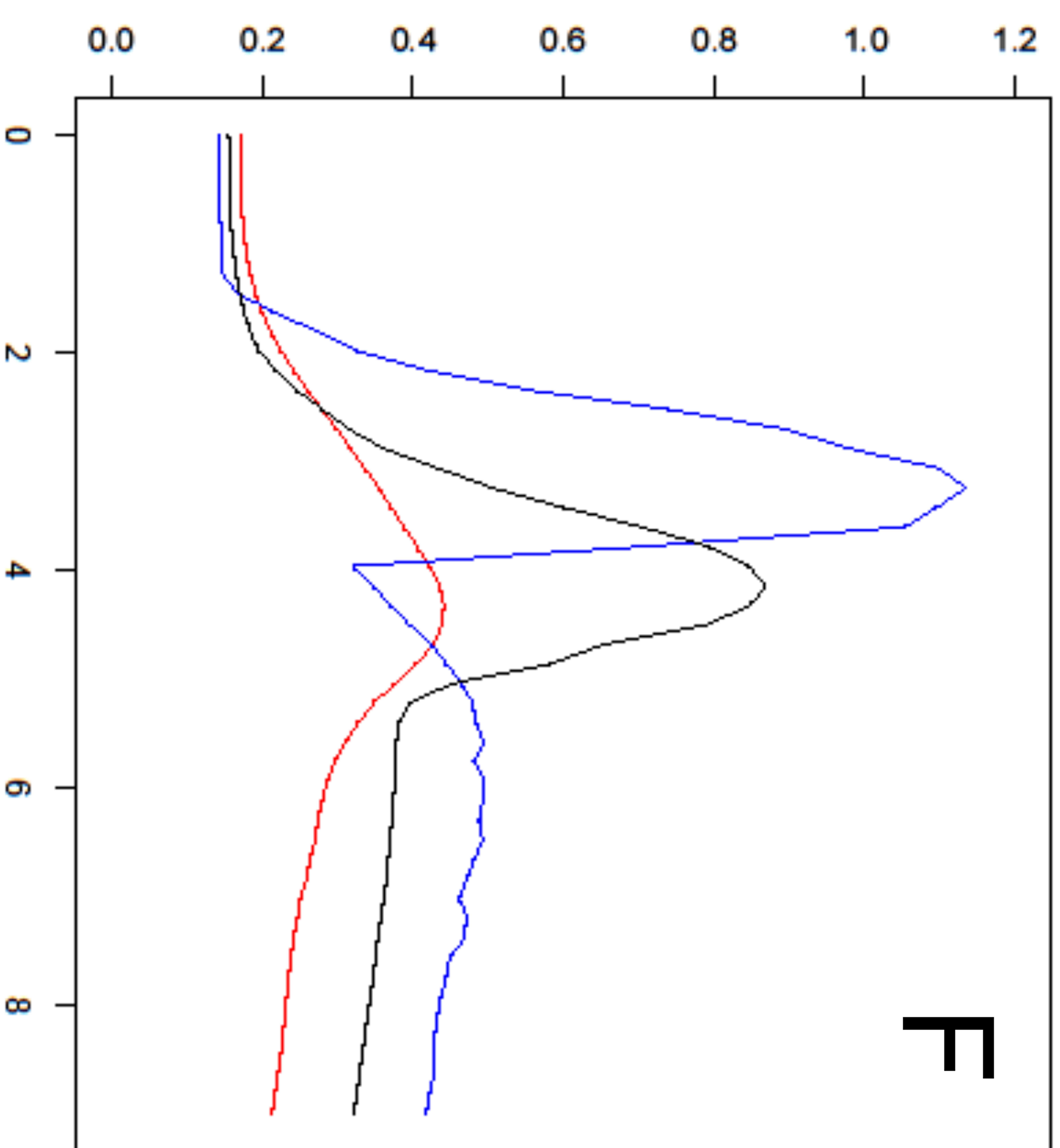

Supplement: Supplementary file 3 — Figure S3. Coefficients of variation of effective population size estimates after exponential expansion. [file eva0007-0663-SD3.pdf]

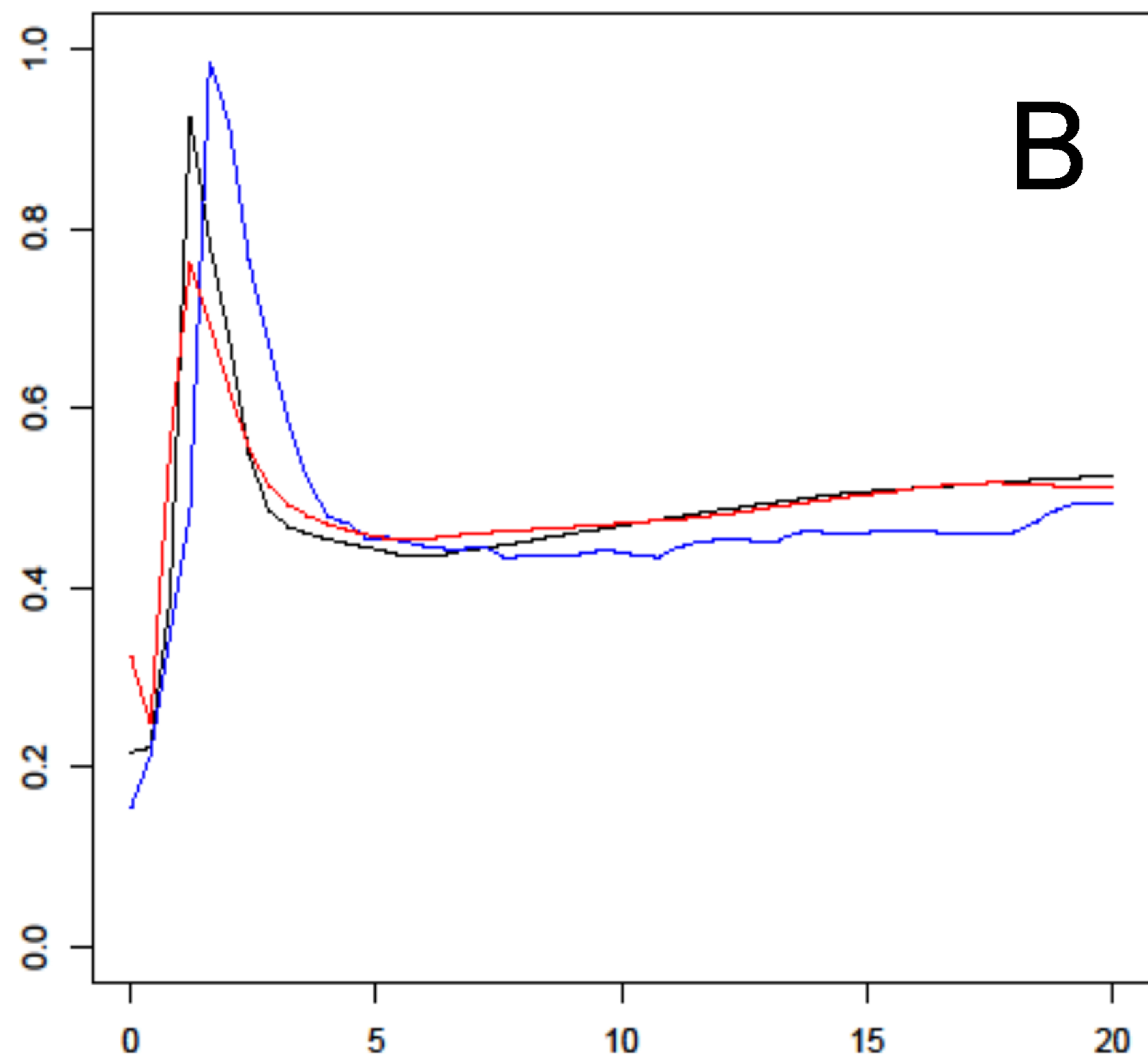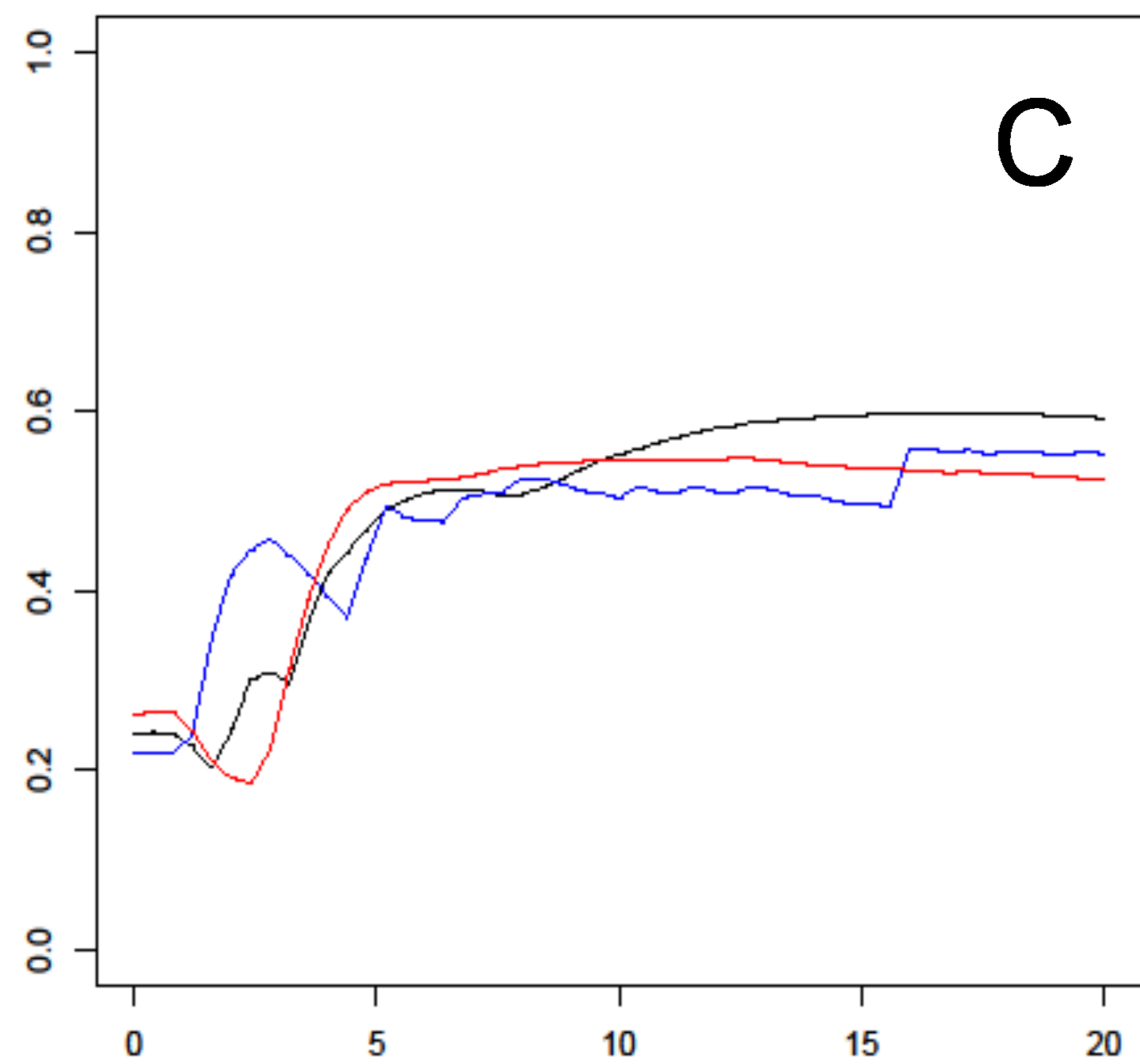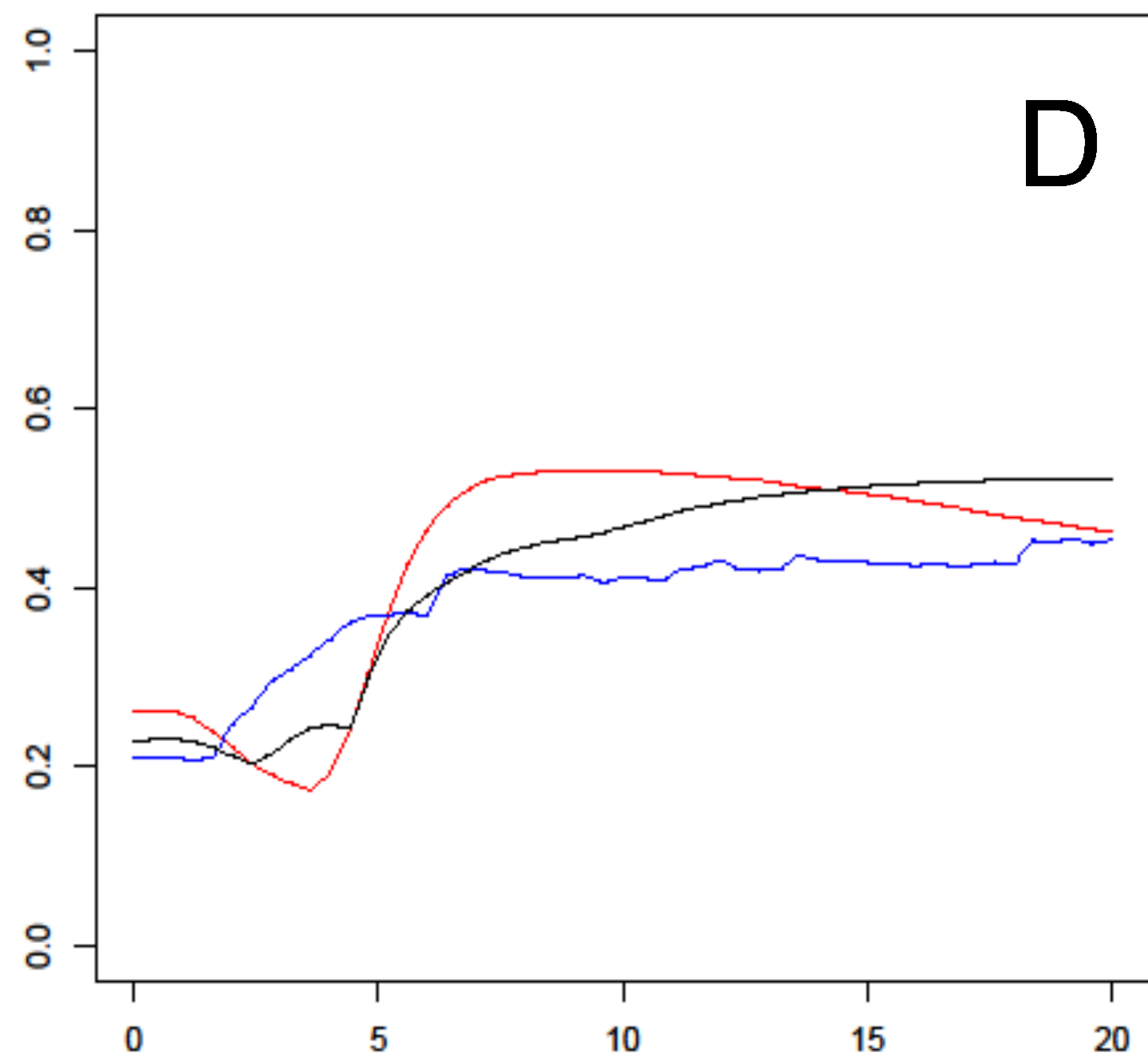

Supplement: Supplementary file 4 — Figure S4. Coefficients of variation of effective population size estimates after a bottleneck. [file eva0007-0663-SD4.pdf]

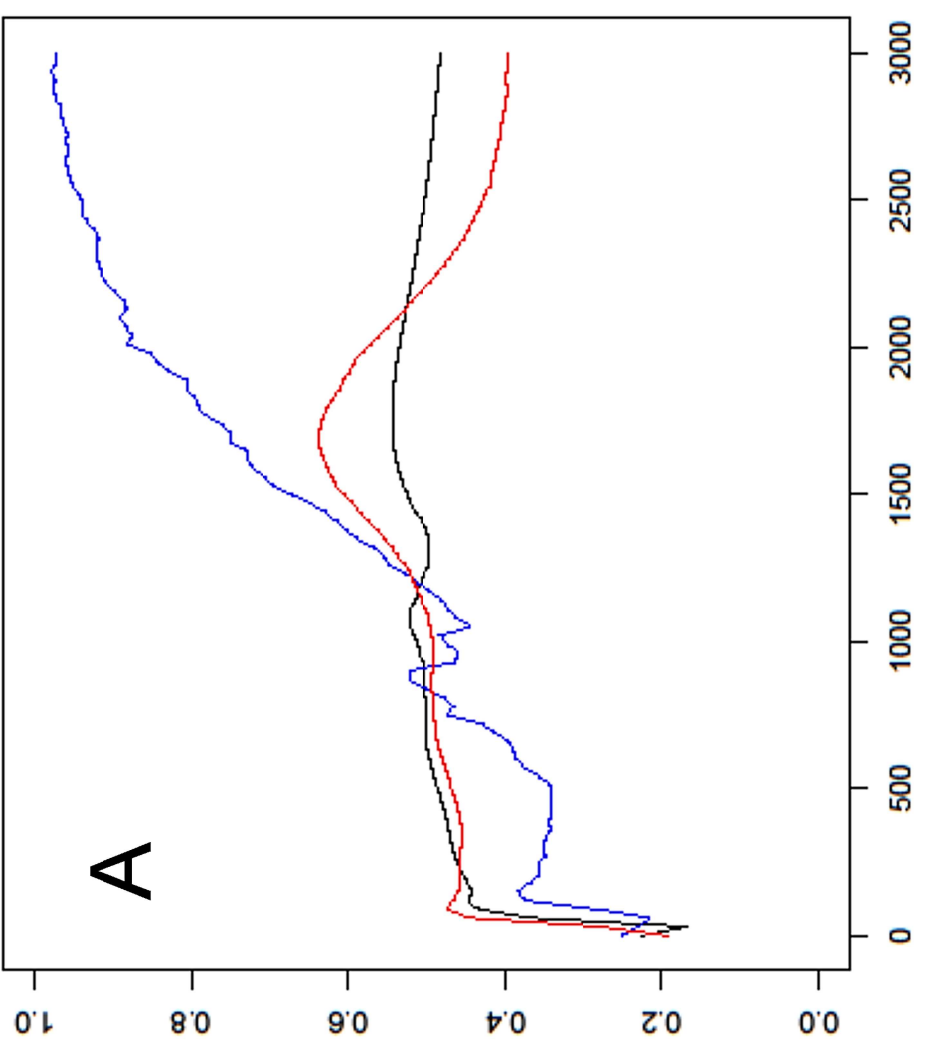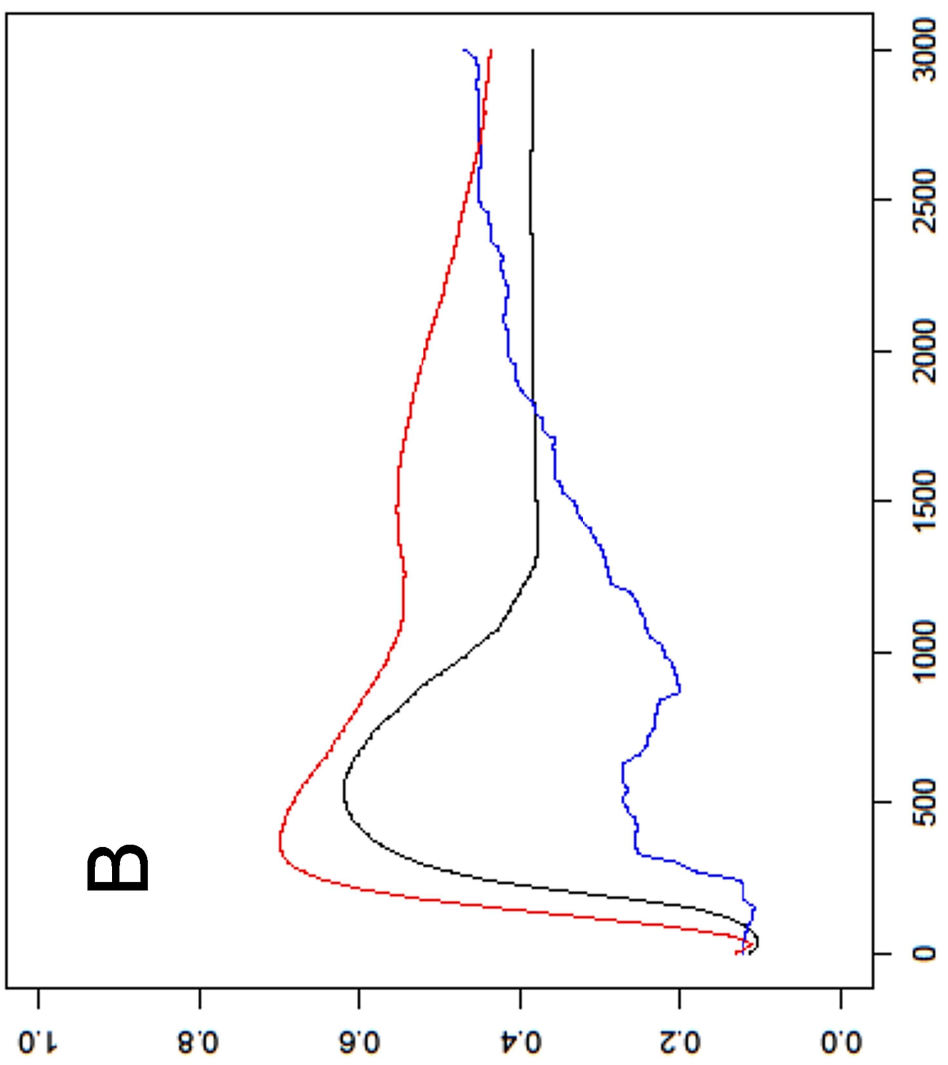

Supplement: Supplementary file 5 — Figure S5. Coefficients of variation of effective population size estimates after a transient increase. [file eva0007-0663-SD5.pdf]
