# Supplementary material for: Detecting past changes of effective population size
Source: Evol Appl. 2014 Jun 16;7(6):663–81. doi: 10.1111/eva.12170 (PMC4105917; doi:10.1111/eva.12170)
Supplement: Supplementary file 6 — Data S1. Genetic diversity at microsatellite markers in a finite population. Data S2. The distribution of the TMRCA in a simple migration model. [file eva0007-0663-SD6.docx]

**Supporting information**

**Data S1.**

**Genetic diversity at microsatellite markers in a finite population**

For a microsatellite marker that follows a stepwise mutation model, each allele is characterized by a number (*i*) of microsatellite motifs, and a distance *D* can be defined between two alleles as the difference *k=i-j* of the numbers of motifs they include. For each mutation event, it is assumed that the allele derived from allele (*i*) may carry *i±r* motifs, with probabilities ½ *m_r_*. An extension of the usual calculation of inbreeding coefficient in finite population can then be carried out, including the specification of allelic diversity. In a population of finite size, one considers the probabilities that a pair of alleles is characterized by a distance *k* and that the time since they have derived from a common ancestor gene (their $T_{MRCA}$ ) is less that some time $\tau$:

$\Pr\left( T_{MRCA}<\tau, D=k \right)$.

Then, one calculates the same expression in the next generation (g+1), which includes terms involving distances *k±r*, probabilities *m_r_* and the size *N(g)* of the population at time (g). Considering a stepwise mutation model, the infinite set of equations written for the different *k* values can be turned into a single functional equation when the characteristic function *F* is introduced (Wehrhahn 1975, Rousset 1996):

$$F\left( \tau,x \right)= \sum_{k=-\infty}^{k=+\infty} \Pr\left( T_{MRCA}<\tau, D=k \right)cos(kx) .$$

If *N* is large, and the mutation rate *µ* is small, so that the product *θ=4Nµ* remains of the order of unity, the recurrence equation in *F* can be turned into an ordinary differential equation,

|  | $\frac{1}{2}\frac{dF(\tau,x)}{d\tau}=\frac{1}{\theta(\tau)}-( \frac{1}{\theta\left( \tau\right)}+\left( 1-M\left( x \right) \right) F\left( \tau,x \right),$ | [A1] |
| --- | --- | --- |

where time is expressed as the product $\tau$ *= gµ,* population sizes as *θ= 4Nµ* values and where *M(x)* is the characteristic function of the stepwise mutation model. Now, provided $\theta\left( \tau\right)$ is a step function, this differential equation can be analytically solved (Chevalet and Nikolic 2010).

**The *f_k_* distribution**

In a sample of alleles, the expectation of the frequency ***f_k_*** of pairs of alleles at distance *D=k* is equal to the probability $\Pr\left( D=k \right).$ Recovering this probability from the $F\left( \tau,x \right)$ function is obtained from the Cauchy inversion formula:

$$Pr(D=k)=\frac{1}{2\pi} \int_{-\pi}^{+\pi} F\left( \infty,x \right) cos(kx) .$$

Since the *F* function is an explicit function of *x* and of the demographic parameters $\left( \boldsymbol{\theta\tau} \right)$, getting the numerical value only needs a numerical integration.

**The distribution of** $\boldsymbol{T}_{\boldsymbol{MRCA}}$

Setting $x = 0$, in Equation A1 ($F(\tau,0) = P(T_{MRCA} < \tau) )$ allows the distribution of $T_{MRCA}$ to be recovered, and this equation shows its link with the function $\theta(\tau)$ describing the change with time of population size:

|  | $\theta\left( \tau\right)= \frac{2(1-F\left( \tau,0 \right))}{\frac{dF(\tau,0)}{d\tau}}$ . | [A2] |
| --- | --- | --- |

**Data S2.**

**The distribution of the T_MRCA_ in a simple migration model**

We consider here a simple migration model from a large population into a population of constant size *N* into which a proportion *m* of gametes are contributed by the large external population, following the approach of Hobolth et al. (2011). We assume that effective population sizes are constant over time, *N* and *N/ε* where the ratio *ε* will be assumed small. In this note we restrict the analysis to the distribution of ***T_MRCA_*** of two random alleles, but, as illustrated by Rousset (1996), the distribution conditional on the distance *k* between microsatellite alleles can be derived in the framework of this migration scheme as well as the expected *f_k_* distribution, and could be used to estimate migration rates together with population sizes.

***T_MRCA_* distribution**

Consider two alleles drawn at present time in the small population, and their ancestors *g* generations before present time. These ancestors may be in 5 exclusive states:

(1) They are confounded into a single allele of the small population because a coalescence event occurred within the small population between present time and the *g*-th generation in the past.

(2) They are two distinct alleles within the small population because all ancestor alleles remained within the population and no coalescence event occurred.

(3) They are two distinct alleles, one within the small population, and one in the large external population, because one immigration event occurred in the ancestral line of one of the alleles.

(4) They are two distinct alleles in the large external population, because both ancestral lines were affected by an immigration event.

(5) They are confounded into a single allele of the large external population because a coalescence event occurred in the large population.

Denoting by $P_{i}^{(g)}$ the probabilities of these five states, we note that $P_{1}^{(g)}+ P_{5}^{(g)}$ is the probability that the time $T_{MRCA}$ to coalescence is less than or equal to *g*. We then derive the backward transition probabilities giving the conditional probabilities *T_ij_* of states *i* at past generation *g+1* if ancestors alleles were in state *j* at time *g*. Neglecting the probabilities that two rare events occur at the same time, we get the following *T* matrix,

|  | $T= \left( \begin{matrix} 1-m & \frac{1}{2N} & 0 & 0 & 0 \\ 0 & 1-2m-\frac{1}{2N} & 0 & 0 & 0 \\ 0 & 2m & 1-m & 0 & 0 \\ 0 & 0 & m & 1-\frac{\varepsilon}{2N} & 0 \\ m & 0 & 0 & \frac{\varepsilon}{2N} & 1 \end{matrix} \right)$ |  |
| --- | --- | --- |

so that $\boldsymbol{P}^{(g+1)}=T . \boldsymbol{P}^{(g)}$ where ***P***'s are vectors of the *P_i_'*s probabilities. Solving with initial condition $P_{2}^{(0)}=1$, we get:

|  | $\Pr\left( T_{MRCA} \leq g \right)=P_{1}^{\left( g \right)}+ P_{5}^{\left( g \right)} =1-A \left( 1-2m-\frac{1}{2N} \right)^{g}-B\left( 1-m \right)^{g}-C\left( 1-\frac{\varepsilon}{2N} \right)^{g}.$ |  |
| --- | --- | --- |

Changing time scale to $t=\frac{g}{2N}$ and introducing the migration parameter $\nu= 2Nm$, we write:

|  | $F\left( t \right)=Pr\left( T_{MRCA} \leq t \right)\simeq1-A\exp\left( -(1+2\nu\right)t)-B{\exp(-\nu t)}-C \exp(-\varepsilon t)$ | [A3] |
| --- | --- | --- |

with:

$A=\frac{1+\nu+ \epsilon(\nu-1)}{\left( 1+\nu\right)(1+2\nu-\varepsilon)}$ ,

$B= - \frac{2\epsilon\nu}{\left( \nu-\varepsilon\right)(1+\nu)}$ , and

$C=\frac{2 \nu^{2}}{\left( \nu-\epsilon\right)(1+2\nu-\varepsilon)}$ .

**Apparent past demography**

Without migration ($\nu$ = 0), the distribution of coalescent time in a population with constant effective size *N* is reduced to the first term in the previous equation with *A = 1* and $\nu$ = 0. In a closed population with changing effective size *N(t)*, Equation A2 gives the link between functions *N(t)* and *F(t)*. With the present scale and notation, this equation reads:

|  | $N\left( t \right)= N\left( 0 \right)\frac{1-F(t)}{dF(t)/dt}$ | [A4] |
| --- | --- | --- |

Plugging Equation A3 into Equation A4 generates an artifactual dependence of population size on time, according to which the size would move from the present actual size *N(0)* to the size *N(0)/ε* of the external larger population that sends immigrants, with a transient burst when *N(t)* exceeds the ultimate large size. For a small value of *ε*, neglecting the B term in Equation A3, the following approximation holds:

|  | $N\left( t \right)\simeq N\left( 0 \right)\frac{2 \nu+\exp(-\left( 1+2\nu-\epsilon\right)t )}{2\nu\epsilon+\left( 1+2\nu\right)\exp(-\left( 1+2\nu-\epsilon\right)t )}$ |  |
| --- | --- | --- |

The zero of the second derivative then provides the time when a sudden apparent change of the past effective size occurred, i.e. the time of an apparent past bottleneck; this time is given by:

|  | $t_{b}=\frac{1}{1+2\nu} \left( \ln\frac{1}{\epsilon} + \ln\frac{1+2\nu}{2\nu} \right)$ | [A5] |
| --- | --- | --- |

Numerical investigations of Equation A5 using the complete expression A3 indicated that this approximation for $t_{b}$ remained qualitatively meaningful.

From this expression, the migration rate *m* may be derived from observed or estimated values of the ratio *ε* and of the age of the apparent bottleneck. Provided $2 \nu= 4Nm$ is not too small (> 10, say), $m \simeq\left( \frac{1}{2}\ln\frac{1}{\epsilon} \right)/{g_{b}}$, where *g_b_* is the age of the bottleneck where time is measured in generations.

**Additional references**

Hobolth A, LN Andersen and T Mailund. 2011. On computing the coalescence time density in an isolation-with-migration model with few samples. Genetics 187:1241-1243.

Rousset F. 1996. Equilibrium values of measures of population subdivision for stepwise mutation processes. Genetics 142:1357-1362.

Wehrhahn C. 1975. The evolution of selectively similar electrophoretically detectable alleles in finite natural populations. Genetics 80: 375-394.

**Legends of Supplementary Figures**

**Figure S1.** Accuracy of population size estimates from the Mode and the Median as function of the diagonal *λ* parameter.

From bottom to top: accuracies observed on *100* replicates from the estimation of population size at past times, from *0* (present time) to *4.5* (reduced time scale). Constant population size was set to *θ= 12* and estimations were based on genotypes at *40* markers. Ordinates: accuracy, as in Figures 1 and S1. Abscissa: *λ* parameter. (A) Mode estimate; (B) Median estimate.

**Figure S2.** Accuracy of the estimation of the current population size from estimates in the past.

Data were generated from *100* simulated populations with constant population size characterized by *θ = 40* and estimations were based on genotypes at *40* markers. Abscissa: times in the past when population size is estimated (reduced scale: generation x mutation rate). Ordinates: relative deviation of estimate from the true value, calculated as the ratio $\sqrt{MSE}/\theta$ of the square root of the Mean Square Error of the estimate to the true *θ* value, for the four measures: arithmetic mean (red diamonds), harmonic means (orange squares), mode (blue triangles), and median (black squares) of the posterior distribution of effective size.

**Figure S3.** Coefficients of variation of effective population size estimates after exponential expansion.

Cases B, D and F are the same as in Figure 2. Abscissa: time in the past, in reduced scale generation x mutation rate, from *0* to *9*. Ordinates: coefficients of variation (ratios of standard deviations to the means) of estimates from arithmetic means (red), medians (black) and modes (blue) of posterior distributions.

**Figure S4.** Coefficients of variation of effective population size estimates after a bottleneck.

Cases B, C and D are the same as in Figure 3. Abscissa: time in the past, in reduced scale generation x mutation rate, from *0* to *20*. Ordinates: coefficients of variation (ratios of standard deviations to the means) of estimates from arithmetic means (red), medians (black) and modes (blue) of posterior distributions.

**Figure S5.** Coefficients of variation of effective population size estimates after a transient increase.

Cases A and B are the same as in Figure 4. Abscissa: time in the past, in generations, from *0* to *3000*. Ordinates: coefficients of variation (ratios of standard deviations to the means) of estimates from arithmetic means (red), medians (black) and modes (blue) of posterior distributions.
